# Glial pathology and retinal neurotoxicity in the anterior visual pathway in experimental autoimmune encephalomyelitis

**DOI:** 10.1186/s40478-019-0767-6

**Published:** 2019-07-31

**Authors:** Jing Jin, Matthew D. Smith, Calvin J. Kersbergen, Tae-In Kam, Mayuri Viswanathan, Kyle Martin, Ted M. Dawson, Valina L. Dawson, Donald J. Zack, Katharine Whartenby, Peter A. Calabresi

**Affiliations:** 1 Department of Neurology, Johns Hopkins University School of Medicine, Baltimore, MD 21205 USA; 2 Neuroregeneration and Stem Cell Programs Institute for Cell Engineering, Johns Hopkins University School of Medicine, Baltimore, MD 21205 USA; 3 Department of Neuroscience, Johns Hopkins University, Baltimore, MD USA; 4 Department of Pharmacology and Molecular Sciences, Johns Hopkins University School of Medicine, Baltimore, MD 21205 USA; 5 Department of Physiology, Johns Hopkins University School of Medicine, Baltimore, MD 21205 USA; 6 Departments of Ophthalmology, The Johns Hopkins University School of Medicine, Baltimore, MD 21205 USA; 7 The Guerrieri Center for Genetic Engineering and Molecular Ophthalmology The Wilmer Eye Institute, The Johns Hopkins University School of Medicine, Baltimore, MD 21205 USA; 8 Division of Neuroimmunology and Neurological Infections Pathology Building, Johns Hopkins Hospital, 627 600 N. Wolfe St., Baltimore, MD 21287 USA

**Keywords:** EAE, Astrocytes, Retinal ganglion cells, Optic neuritis

## Abstract

The animal model experimental autoimmune encephalomyelitis (EAE) has been used extensively in the past to test mechanisms that target peripheral immune cells for treatment of multiple sclerosis (MS). While there have been some notable successes in relapsing MS, the development of therapies for progressive multiple sclerosis (MS) has been hampered by lack of an appropriate animal model. Further, the mechanisms underlying CNS inflammation and neuronal injury remain incompletely elucidated. It is known that the MOG _35–55_ EAE mouse model does not have insidious behavioral progression as occurs in people with MS, but there is significant neuronal and axonal injury in EAE, as a result of the inflammation. In the present study, we describe the time course of glial activation and retinal neurodegeneration in the EAE model, and highlight the utility of studying the anterior visual pathway for modeling mechanisms of neuronal injury that may recapitulate critical aspects of the pathology described in people with MS following optic neuritis and subclinical optic neuropathy. We show that A1 neurotoxic astrocytes are prevalent in optic nerve tissue and retina, and are associated with subsequent RGC loss in the most commonly used form of the EAE model induced by MOG _35–55_ peptide in C57/B6 mice. We developed a semi-automatic method to quantify retinal ganglion cells (RGC) and show that RGCs remain intact at peak EAE (PID 16) but are significantly reduced in late EAE (PID 42). Postsynaptic proteins and neurites were also compromised in the retina of late EAE mice. The retinal pathology manifests weeks after the microglial and astrocyte activation, which were prominent in optic nerve tissues at PID 16. Microglia expressed iNOS and had increased gene expression of C1q, TNF-α, and IL-1α. Astrocytes expressed high levels of complement component 3 and other genes associated with A1 neurotoxic astrocytes. Our data suggest that EAE can be used to study the pathobiology of optic neuropathy and to examine the preclinical neuroprotective effects of drugs that target activation of neurotoxic A1 astrocytes.

**Electronic supplementary material:**

The online version of this article (10.1186/s40478-019-0767-6) contains supplementary material, which is available to authorized users.

## Introduction

The mechanisms underlying neurodegeneration remain incompletely defined for most progressive neurological diseases, including multiple sclerosis (MS). Animal models that recapitulate aspects of human disease are necessary to both understand the in vivo mechanisms of neuronal death and to screen neuroprotective drugs, but no single model accurately reflects the complexity of MS [24]. Experimental autoimmune encephalomyelitis (EAE) is a widely used animal model for examining mechanisms of T-cell activation and trafficking and for testing drugs that may be useful in MS [38]. However, screening for novel anti-inflammatory agents in EAE has been scrutinized because numerous drugs broadly inhibit peripheral immune cell pathways and thereby prevent or treat acute EAE, but these approaches are not specific for immune mechanisms involved in MS and therefore render patients susceptible to infections related to immune suppression [7]. Further, many agents can suppress inflammation that precedes or occurs during peak behavioral deficits, akin to an MS relapse, but have little effect on mechanisms that ensue within the central nervous system (CNS) and mediate subsequent neurotoxicity, much in the way that corticosteroids shorten the duration and severity of a relapse but appear to have little effect on long-term outcomes [4, 37]. There remains a need to study the mechanisms underlying CNS inflammation that differ from peripheral targets or edema. The roles of microglia and astroglia have long been studied in MS and related animal models, and recently transcriptomic profiling of glia has allowed for more specific target identification for neuroprotection [5, 6, 13, 17, 21, 40]. We sought to better define the profiles of activated glia and the time course of neurotoxicity [3, 8, 25, 29, 31, 52] in EAE, and to develop quantitative methods for measuring neurodegeneration. We and others have previously shown that in EAE there is extensive loss of axons in the spinal cord following an acute wave of immune cell infiltration into the CNS [18, 53]. Proper axon quantification in the spinal cord is laborious and is complicated by the multiple ascending and descending fibers and the distance from their associated cell bodies neurons. Alternatively, the anterior visual pathway affords the opportunity to quantify pathology in the optic nerve, another primary location of immune cell infiltration in EAE, as well as in the retina to assess retinal ganglion cell (RGC) loss and local microglia and astroglia responses [14, 15, 23, 34, 46, 47]. There is also an extensive literature linking optic neuropathy in MS to retinal ganglion cell layer thinning as measured with optical coherence tomography (OCT), and the extent of thinning mirrors global cerebral atrophy and predicts disability outcomes [28, 33, 41–45, 49, 51]. Thus, quantifying RGC loss and elucidating the glial mechanisms of injury has great translational potential. Herein, we have developed a semi-automated algorithm to quantify RGC cell number on retinal flat mounts for high-throughput analysis of neurodegeneration. In a mouse EAE model induced with myelin-oligodendrocyte glycoprotein peptide 35–55 (MOG_35–55_), we found significant RGC loss in the retina of late EAE mice, post-immunization day (PID) 42, but not in early EAE (PID 16). RGC loss was observed in association with reduced post-synaptic proteins and neurite projections, which were accompanied by persistent astroglial activation in the inner retina. Optic neuritis was evident at the early stage with marked activation of microglia and astroglia. The activated microglia expressed high levels of iNOS, and optic nerve tissues had higher mRNA level for the cytokines IL-1α, TNF-α, and C1q, which were recently implicated in activation of neurotoxic astrocytes [27]. Indeed, the astrocytes from hindbrain of EAE mouse had increased neurotoxic astrocyte gene expression (A1-transcripts), but decreased neuroprotective astrocyte gene expression (A2-transcripts) [27]. Increased astrocyte A1-transcripts were confirmed in peak EAE optic nerve tissue as well, with reduced A2-transcripts. Immunohistological staining further confirmed that in the optic nerve astrocytes were skewed to the neurotoxic subtype, which expressed high levels of complement component 3 (C3) and a neurotoxic astrocyte marker, immunoproteasome subunit beta type-8 (PSMB8).

## Material and methods

### Animals

C57BL/6 J (B6) mice were purchased from The Jackson Laboratory. All animals were housed in the pathogen-free, temperature controlled animal facility at the Johns Hopkins University School of Medicine with 12 h/12 h light/dark cycles and fed with standard food and water ad libitum. All experimental protocols were performed in accordance with the National Institutes of Health guidelines for the use of experimental animals and were approved by the Johns Hopkins Institutional Animal Care and Use Committee.

### EAE model

EAE was induced in wild-type C57Bl/6 J mice following previous protocols [10]. All studies were carried out using 7–8-week-old mice. Mice were injected subcutaneously with 150 μg of MOG_33–55_ (Johns Hopkins Peptide Synthesis Core) in complete Freund’s adjuvant (CFA) (ThermoScientific, Rockford, IL, USA) containing 600 μg of *Mycobacterium tuberculosis* (BD, Franklin Lakes, NJ) on the lateral abdomen. On days 0 and 2 of disease course, mice were also injected IP with 375 ng of pertussis toxin (List Biological Labs, Campbell, California, USA). Mice were sacrificed at peak disease, PID 16 and late stage, PID 42. For controls the same protocol was used but mice were immunized with CFA without peptide.

Clinical EAE behavioral scores were graded daily, in a masked manner, using the established standard scoring from 1 to 5 as follows: 0, no signs of disease; 1, loss of tail tonicity; 2, loss of tail tonicity and mild paralysis of hindlimbs; 3, paralysis of hindlimbs; 4, hindlimb paralysis and mild paralysis of forelimbs; and 5, complete paralysis or death [10].

### Mouse eyeball and optic nerve sample preparation

Mice were injected IP with sodium pentobarbital (100 mg/kg body weight) and cardiac perfused with 30 mL phosphate buffered saline (PBS). After perfusion, the eyes were removed from the eye socket by blunt enucleation using curved dressing forceps and immediately placed into 4% paraformaldehyde (PFA) for immersion fixation for 3 h then transferred into 30% sucrose for cryoprotection.

Whole retinas were dissected from the eyes and four even cuts were made to create a flat retina, which was then processed for RGC staining. After staining, the whole retinas were flat-mounted onto slides and imaged for RGC counting. For cross sectional tissue analyses, the eyes were placed into TissueTek mold filled with Optimal Cutting Temperature compound (O.C.T.) (VWR, Radnor, PA, USA) and snap frozen with 2-methylbutane and then stored at − 80 °C until cryosectioned. The eyes were sectioned at 16 μm vertically and then mounted onto slides (ThermoScientific, Rockford, IL, USA) followed by staining with antibodies.

Optic nerves were dissected out and evenly divided into three parts and then vertically placed into TissueTek mold filled with O.C.T. and snap frozen with 2-methylbutane. Frozen samples were stored at − 80 °C until cryosectioning. Optic nerves were sectioned at 10 μm thickness and then mounted on slides for further immunofluorescence staining.

### Immunofluorescence staining

**Fig. 1:**
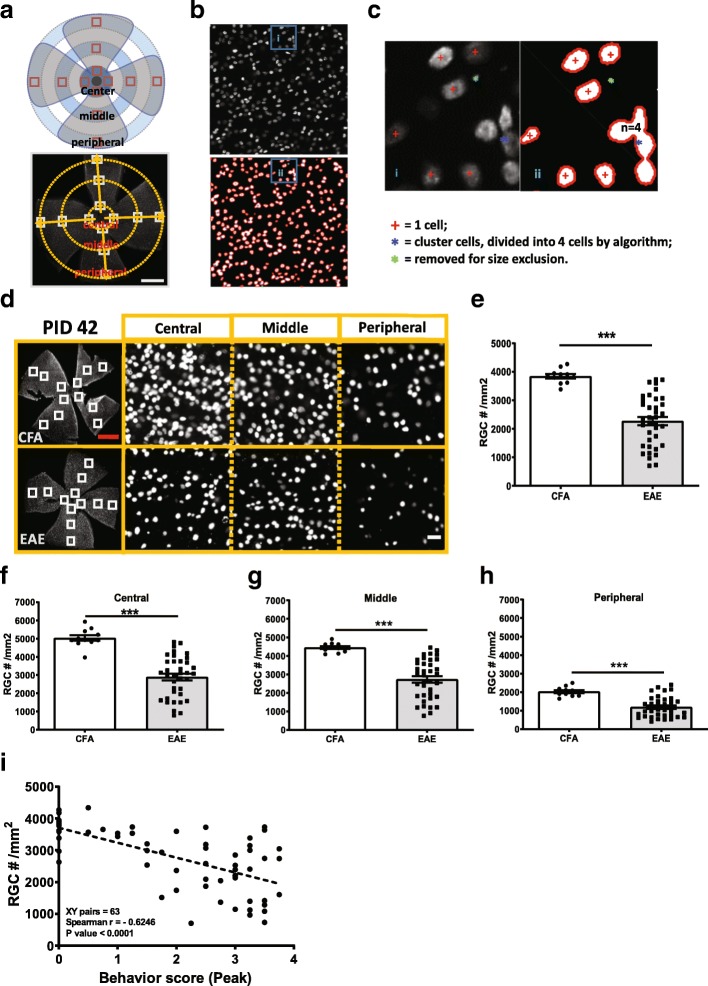
EAE mice had significant RGC loss at late stage but not early stage (peak) of disease. Mouse RGCs were labeled with anti-mouse Brn3a antibody in whole flat mount retina and RGC number was quantified using a MATLAB based semi-automatic algorithm. **a** Schematic of segments selection in whole flat mount retina stained with anti-mouse Brn3a antibody. Scale bar = 1 mm. **b** Cells in selected retinal segment was identified using semi-automatic algorithm. Cell boundaries were identified by red lines, and single cells identified by red “+” symbol; any remaining noise was eliminated by size exclusion, as indicated by green dots. Clusters of overlapping cells, identified by blue dots, were segmented based on the rounded area of the cluster relative to the average cell size. **c** Details of cell identification in Fig. S1c as indicated in boxes. **d** Brn3a staining at PID42. **e-h** Quantification of RGC number at PID42 (**e**), and subregion of whole retina, central (**f**), middle (**g**) and peripheral (**h**). **i** Correlation of RGC numbers and peak behavior score (Spearman *r* = − 0.6246, *p* < 0.001). Error bars represent standard error. Significance of mean differences was compared by two-tailed, unpaired Student’s t-test with *P* < 0.05 considered as significant. *** *P* ≤ 0.001. N.S. = no significant difference. Correlation anylasis was performed by Spearman r test. Red scale bar =1 mm. White scale bar =20 μm

**Fig. 2:**
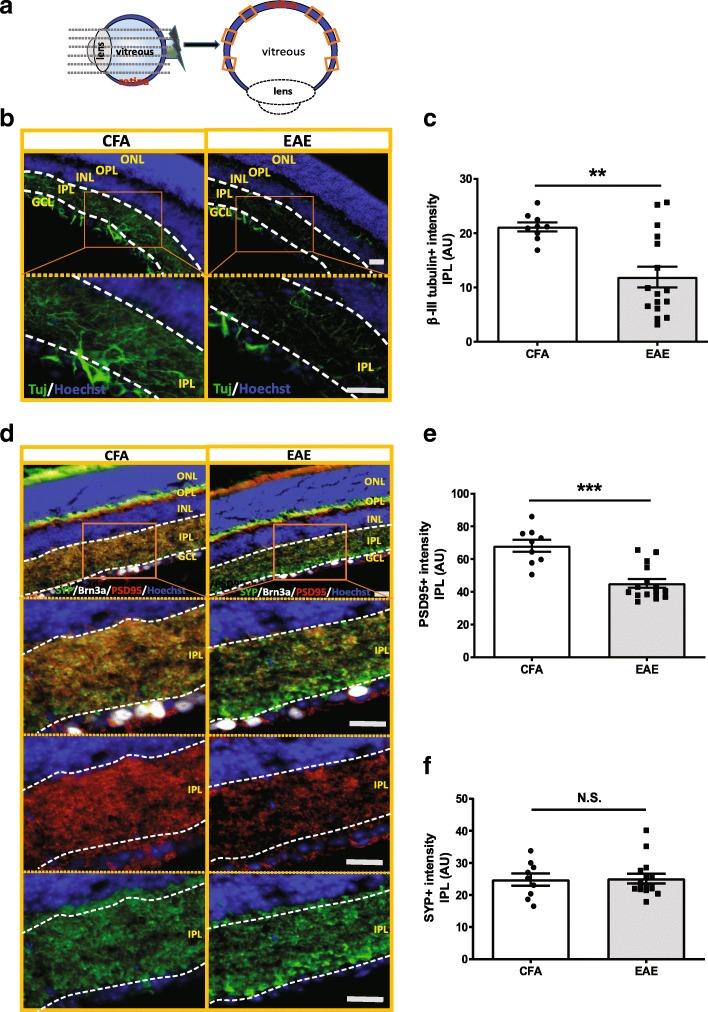
Neurite projection and synaptic density were lost in inner retina of EAE mice at PID42. **a** Schematics for mouse eyeballs verticle section and retinal region selection. **b** Neurite projections in inner retina was stained with β-III-tubulin (Tuj). **c** Quantification of Tuj positive neurites in the inner plexiform layer (IPL) of retina in EAE mice (*n* = 17) vs CFA control mice (*n* = 9). **d** Presynaptic marker, synaptophysin (SYP), and the postsynaptic marker, PSD95 staining in CFA and EAE mouse retina. **e**, **f** Quantification of PSD-95 (**e**) and SYP (**f**) staining intensity in the inner retina of EAE mouse (*n* = 15) and CFA control (*n* = 9). Quantification of Tuj, SYP and PSD95 is represented as mean ± SEM. Significance was determined by two-tailed, unpaired Student’s t-test with *P* < 0.05 considered signficant. ** *P* ≤ 0.01. N.S. = no significant difference. Scale bar =20 μm

Sections were permeabilized with PBS containing 0.4% of Triton X-100 then blocked with PBS containing 5% normal donkey serum and 0.1–0.3% Triton X-100 for 1 h at room temperature and incubated with primary antibody of interest overnight at 4 °C. The primary antibodies that were used are shown in Table 1. The sections were then incubated for 1 h at room temperature with species-specific secondary antibodies directly conjugated to Alexa fluorophores (1:1000, Invitrogen) followed by nuclei staining with Hoechst. A coverslip was mounted onto sections using aqua poly/mount reagent (Polysciences, Warrington, PA, USA). Images were captured using a Zeiss Axio Observer Z1 epifluorescence microscope and Axiovision software with the appropriate excitation and emission filters. For analyzing the flat mount retinal preparation, 12 locations were used to determine regional RGC counts (Fig. 1a and Additional file 1: Figure S1b); regions of interest (ROI) (delineated by white dashed lines in figure) of six areas from each retina were analyzed as shown in Fig. 2a; and nine sections of optic nerve were analyzed from three regions as in Fig. 4a. ImageJ software (National Institutes of Health) was used to create a binary image of the staining and subsequently quantify staining intensity by persons blind to sample information. For enumerating glia and T cells, Zen Blue software (Carl Zeiss, Oberkochen, Germany) was used.

**Table 1:** Primary antibodies used for immunoflurorescence staining

Antibody	Source	Dilution
Mouse anti-NeuN	Millipore	1:100
Goat anti-Brn3a	Santa Cruz Biotech	1:100
Rabbit anti-RBPMS	GeneTex	1:100
anti-β-III tubulin	BD Pharmingen	1:100
anti-Synaptophysin	Millipore	1:100
Anti-PSD-95	ThermoFisher Scientific	1:200
Rabbit anti-IBA-1	Wako	1:500
Rabbit anti-GFAP	DAKO	1:500
Rat anti-C3	Hycult Biotech	1:200
Rat anti-CD4	Biolegend	1:100
Mouse anti-SMI31	Biolegend	1:250
Mouse anti-SMI32	Biolegend	1:250
Rabbit anti-MBP	Cell Signaling Technology	1:250
Mouse anti-GFAP	Cell Signaling Technology	1:250 ON, 1:500 Retina
Anti-iNOS	Santa Cruz Biotech	1:1500
Anti-PSMB8	Abcam	1:100
Rat anti-MAC2	Biolegend	1:100

### RNA extraction and quantitative PCR

Optic nerve total RNA was extracted using Trizol reagent (Sigma, Saint Louis, MO, USA) as recommended by the manufacturer. First-strand cDNA was synthesized using iScript cDNA Synthesis Kit (Bio-Rad, Hercules, California, USA). Quantitative PCR was carried out using iTaq SYBRMix (Bio-Rad, Hercules, California, USA) and a CFX384 Touch Real-Time PCR Detection System (Bio-Rad, Hercules, California, USA). Primers were designed using Primer3Plus or from published resources [27]. Table 2 shows the specific primer sequences used. All target genes were normalized to actin mRNA levels and fold change was calculated by performing delta-delta Ct analysis.

**Table 2:** Primers for qPCR

primers	Forwards sequence 5′ -- 3′	Reverse sequence 5′ -- 3′
*β-Actin*	ACCTTCTACAATGAGCTGCG	CTGGATGGCTACGTACATGG
*TNFα*	TGTGCTCAGAGCTTTCAACAA	CTTGATGGTGGTGCATGAGA
*IL-1α*	CGCTTGAGTCGGCAAAGAAAT	CTTCCCGTTGCTTGACGTTG
*C1q*	AGACACAGTGGGGTGAGGTC	GGTCCCCTTTCTCTCCAAAC
*C3*	AGCTTCAGGGTCCCAGCTAC	GCTGGAATCTTGATGGAGACGC
*H2-T23*	GGACCGCGAATGACATAGC	GCACCTCAGGGTGACTTCAT
*H2-D1*	TCCGAGATTGTAAAGCGTGAAGA	ACAGGGCAGTGCAGGGATAG
*PSMB8*	CAGTCCTGAAGAGGCCTACG	CACTTTCACCCAACCGTCTT
*Iigp1*	GGGGCAATAGCTCATTGGTA	ACCTCGAAGACATCCCCTTT
*GBP2*	GGGGTCACTGTCTGACCACT	GGGAAACCTGGGATGAGATT
*SPERPING 1*	ACAGCCCCCTCTGAATTCTT	GGATGCTCTCCAAGTTGCTC
*Ggta1*	GTGAACAGCATGAGGGGTTT	GTTTTGTTGCCTCTGGGTGT
*Fbln5*	CTTCAGATGCAAGCAACAA	AGGCAGTGTCAGAGGCCTTA
*Ugt1a*	CCTATGGGTCACTTGCCACT	AAAACCATGTTGGGCATGA
*Sphk1*	GATGCATGAGGTGGTGAATG	TGCTCGTACCCAGCATAGTG
*CLCF1*	CTTCAATCCTCCTCGACTGG	TACGTCGGAGTTCAGCTGTG
*Ptgs2*	GCTGTACAAGCAGTGGCAAA	CCCCAAAGATAGCATCTGGA
*Ptx3*	AACAAGCTCTGTTGCCCATT	TCCCAAATGGAACATTGGAT
*S100a10*	CCTCTGGCTGTGGACAAAAT	CTGCTCACAAGAAGCAGTGG

### Astrocyte isolation and quantitative PCR

Astrocyte from the hindbrains of peak EAE mice were purified by iodixanol density gradient fractionation as previously described with minor modifications [19]. In brief, the hindbrains were dissected and pulverized to a homogenous powder under liquid nitrogen. After dissociation with trypsin, the cells were resuspended in 6 ml of DMEM with 10% FBS. Each 1 ml of Optiprep (Sigma, Saint Louis, MO, USA) gradient (35, 25, 20 and 15%) were overlaid by 6 ml of the crude cell fraction and centrifuged at 800 g for 15 min in room temperature. The isolated astrocytes, banded across the 25 and 20% interface, were collected and total RNA was extracted using the RNeasy Plus kit (Qiagen, GmbH, Germany). RNA concentration was measured spectrophotometrically using NanoDrop 2000 (ThermoScientific, Rockford, IL, USA). 1–2 μg of total RNA were reverse-transcribed to cDNA using the High-Capacity cDNA Reverse Transcription System (Life Technologies, ThermoScientific, Rockford, IL, USA). Comparative quantitative RT-PCR (qPCR) was performed for each sample with ViiA 7 Real-Time PCR System (Applied Biosystems, ThermoScientific, Rockford, IL, USA) using fast SYBR Green Master Mix (Life Technologies, ThermoScientific, Rockford, IL, USA). The expression levels of target genes were normalized to the expression of β-actin and calculated based on the comparative cycle threshold Ct method (2^-ΔΔCt^). PAN-, A1 specific-, and A2-specific transcripts were analyzed as previously described [54].

### RGC counts

RGC counting was performed as previously published with some modification [26]. Briefly, a mosaic image of the whole mount retina was captured using a Zeiss Axio Observer Z1 epifluorescence microscope with a motorized stage and a z-stack step size of 3 μm. After the images were acquired, each retinal quadrant was segmented into central, middle, and peripheral regions as shown in Additional file 1: Figure S1b. Selected segments were exported as tagged image file format (TIF) and fed into a custom MATLAB (Mathworks, Natick, MA, USA) size segmentation algorithm to perform automated counts. Each segment was approximately 350 × 350 μm^2^. Figure 1a-c show details of the semi-automated quantification of RGC density. Cells in selected retinal segment undergo filtering, automatic or user-defined thresholding, and binarization followed by identification of cell boundaries. Details of cell definition are shown in Fig. 1d. Labeled RGCs were counted at 20 X magnification and a total of 12 regions were averaged to represent RGC density for each mouse retina (Fig. 1a, b and d). All analyses were performed masked to genotype and behavior score and compared to manual counts (Additional file 7).

### Statistical analysis

Statistical analysis was conducted using GraphPad Prism software (GraphPad, San Diego, CA, USA). Two-tailed Student’s *t* test and One-way ANOVA with Tukey’s post hoc test were used to analyze data. Results were considered significant if the *p* value was < 0.05. Error bars indicate SEM in all figures. Spearman’s rank order test was used to analyze the correlation of RGC number with behavior score.

## Results

### Semi-automated quantification of retinal flat mounts in EAE

We developed a semi-automated algorithm to quantify total and regional RGC numbers using a customized MATLAB script. We first assessed RGC labeling with three different antibodies used frequently in RGC quantification in flat mount retinas, RNA-binding protein with multiple splicing (RBPMS), brain-specific homeobox protein 3A (Brn3A, also known as POU4F1) and neuronal nuclei (NeuN). All three antibodies gave similar results for RGC layer neuronal staining (Additional file 1: Figure S1a). However, as the anti-Brn3a antibody provided the most distinct neuronal staining pattern for downstream quantification and had the least background, we utilized this antibody for further RGC quantification. To quantify RGC number in flat mount retinas efficiently, we combined user segmentation with threshold definition and background deduction using customorized MATLAB software. Then Brn3a labeled RGCs were counted at 20 X magnification in four quadrants of the retina in three different regions (central, middle and peripheral) as depicted in Fig. 1a. Brn3a + cells, as visualized by microscopy, were delineated for enumeration as shown in Fig. 1b. Rare clusters were accurately parced by the algorithm (Fig. 1c).discrepancy between manual and semi-automated counting was compared and with no significance, for total or regional counts (Additional file 1: Figure S1b and c). Importantly, the algorithm performed well in naïve retinas and across a spectrum of diseased EAE retinas (Additional file 1: Figure S1b and c). The variability of left and right EAE eye pathology was minimal (Additional file 1: Figure S1b-d) allowing for the contralateral eye to be used for cross-sectional tissue analyses or other downstream applications. We also compared the RGC results counted by different persons on the same retinas. RGC number was consistent within the same retina, one example of comparison is shown in Additional file 1: Figure S1e.

### RGC neuronal degeneration in late, but not peak, EAE

RGCs were enumerated at peak (PID 16) and late EAE (PID 42) and compared to CFA controls at the same time points. While no significant differences in total or regional RGC numbers were seen at peak disease (EAE = 3566 ± 65 cells/mm^2^ with *n* = 11; CFA = 3684 ± 89 cells/ mm^2^, *n* = 8) (Additional file 2: Figure S2 a-e), late EAE retinas had an average of 43.0% fewer RGCs than CFA controls (2272 ± 146/mm^2^ vs 3683 ± 127/mm^2^) (Fig. 1d and e). RGC counts were greatest in the central region and diminished towards the periphery, which is consistent with normal anatomy, but the percent reduction in EAE vs CFA animals at PID 42 was similar for each region (Fig. 1f-h). No differences were seen in RGC counts between CFA control and naïve retina (Additional file 2: Figure S2f), but since CFA may cause CNS glial activation we continued to use CFA as our control for pathologic analyses [35]. The severity of the disease, as measured by behavioral score (BS), was significantly correlated with RGC loss (Fig. 1i). Since non-sick or minimally affected mice have minimal pathology, we only compared EAE mice with BS greater than or equal to 2 with CFA for all pathological outcomes. No differences were seen in severity of EAE between males and females (Additional file 2: Figure S2 g).

### Degeneration of retinal neurites and synapses in late EAE

**Fig. 3:**
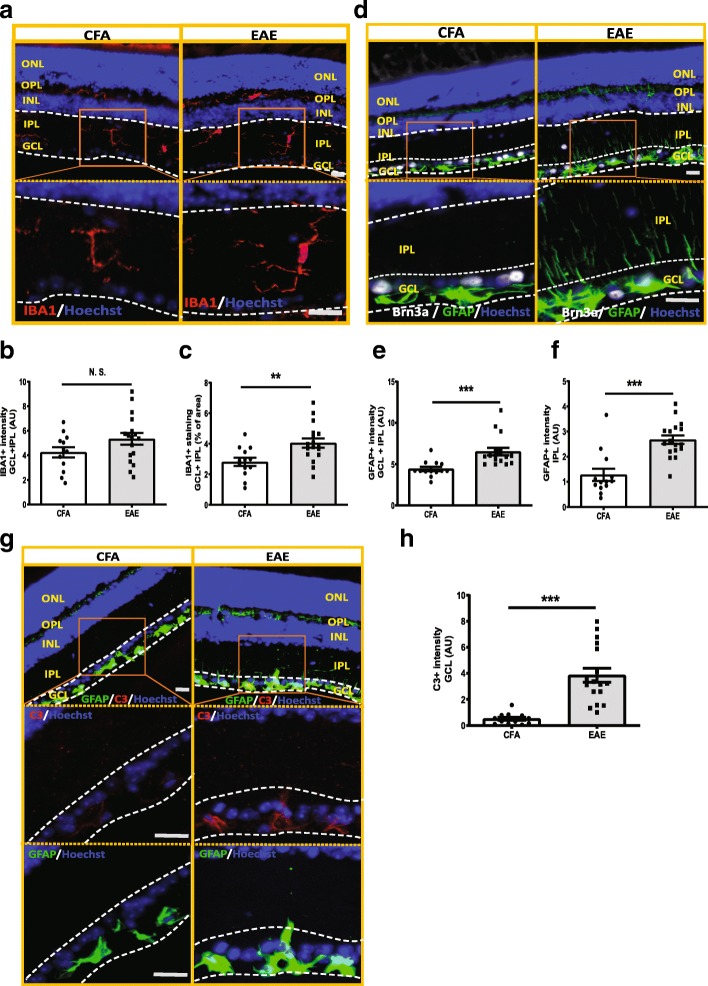
Glial cells were activated in the inner retina of EAE mouse, with increased complement component 3 (C3) activation in GFAP+ glia cells. **a** IBA1 staining in inner retina of EAE mice. In CFA control mice, IBA1+ microglia cells were located in the inner retina with slim processes, distributed in GCL layer, deep IPL layer and OPL layer. However, in EAE retinas, IBA+ cells had more round ameba-liked shape and less ramified morphology, indicating activation of microglial (**a**). **b-c** Quantification of IBA1 staining in EAE (*n* = 17) and CFA control mice (*n* = 13) in GCL and IPL layer of the inner retina. **d** GFAP staining in inner retina of EAE mice. In CFA control mice, GFAP+ cells were mostly located in the GCL and slightly in the OPL. However, in EAE mice retinas, GFAP+ glia cells had longer processes, which extended from the GCL into the deeper IPL (**d**). **e-f** Quantification of GFAP staining in the inner retina of EAE (*n* = 17) and CFA (*n* = 13) control mice in GCL and IPL layer (**e**) and IPL layer (**f**) only. **g** C3 and GFAP double staining in retina of EAE and CFA control mice. **h** Quantification of C3 staining in GCL layer of EAE (*n* = 17) and CFA (*n* = 13) control mice. In EAE mice, GFAP+ cells had higher C3 expression in the RGC layer compared with CFA control mice. Quantification is shown as mean ± SEM. Significance was determined by two-tailed, unpaired Student’s t-test with *P* < 0.05 considered as significant. ** *P* ≤ 0.01, *** *P* ≤ 0.001. N.S. = no significant difference. Scale bar =20 μm

In order to further characterize the associated neuronal retinal pathology at late stage EAE, we examined RGC neurites and synaptic density by staining the retinas with neuron specific β-III-tubulin (Tuj), synaptophysin (SYP) as a presynaptic marker, and postsynaptic density-95 (PSD95) as a postsynaptic marker (Fig. 2). We quantified expression of these markers via immunohistochemistry in six cross-sectional preparations of the retinas of the contralateral eyes not used for RGC counting to examine pathology in the different layers of the retina (Fig. 2a). Tuj positive neurites were significantly decreased in the inner plexiform layer (IPL) of the retina in EAE mice vs CFA control mice (Fig. 2b and c) at PID 42. Consistent with RGC loss, post-synaptic density was decreased in the inner retina of EAE mice, as indicated by reductions in both PSD95 staining intensity and the area of staining in the IPL in EAE mice vs CFA control (Fig. 2d and e and Additional file 3: Figure S3 a). Although the staining intensity and area of SYP, a presynaptic marker from inner nuclear layer neurons adjacent to the RGC, were not different between EAE and CFA (Fig. 2d and f, and Additional file 3: Figure S3 b), the staining pattern of SYP in EAE mice was different from those of CFA control. EAE mice had strong patchy SYP staining in the regions close to the RGC layer, while CFA controls had evenly distributed SYP staining (Fig. 2d). Brn3a + RGCs were also deceased in vertically sectioned retinas in EAE mice as well (Figs. 2d and 3d), consistent with the flat mount retina analysis (Fig. 1e).

### Activation of glia cells in the inner retina in late EAE

In CFA control retinas, ionized calcium-binding adapter molecule 1 (IBA1) positive microglia had slim processes and were located in the GCL, deep IPL and outer plexiform layer (OPL). In EAE retinas, IBA^+^ cells had round amoeboid-like shapes and reduced ramified morphology suggestive of an activated state. Although the staining intensity was not different between EAE and CFA control mice, IBA^+^ signal occupied more area in the GCL and IPL layers of the inner retina in EAE mice (Fig. 3a-c). However, the number of IBA1+ cells was not different between the two groups (Additional file 3: Figure S3c). Glial fibrillary acidic protein (GFAP)-positive glia cells were also activated in EAE mouse inner retina. In CFA control mice, GFAP^+^ cells were mostly located in the GCL layer, but there was also faint staining evident in the OPL layer. In EAE mice, GFAP^+^ glia cells had stronger GFAP expression and there was GFAP staining on processes, which extended from the GCL into the deep IPL (Fig. 3d-f), indicating activation of radial glia (Müller glia), in addition to the resident glia of the GCL [1, 20, 22]. While CFA control mice had no expression of early complement factor 3 (C3), which was recently associated with a neurotoxic astroglia phenotype [27], EAE GFAP^+^ glia had elevated C3 expression, especially in the RGC layer (Fig. 3g and h).

### Infiltration of CD4+ T-cells is present and greater in early EAE vs late EAE

**Fig. 4:**
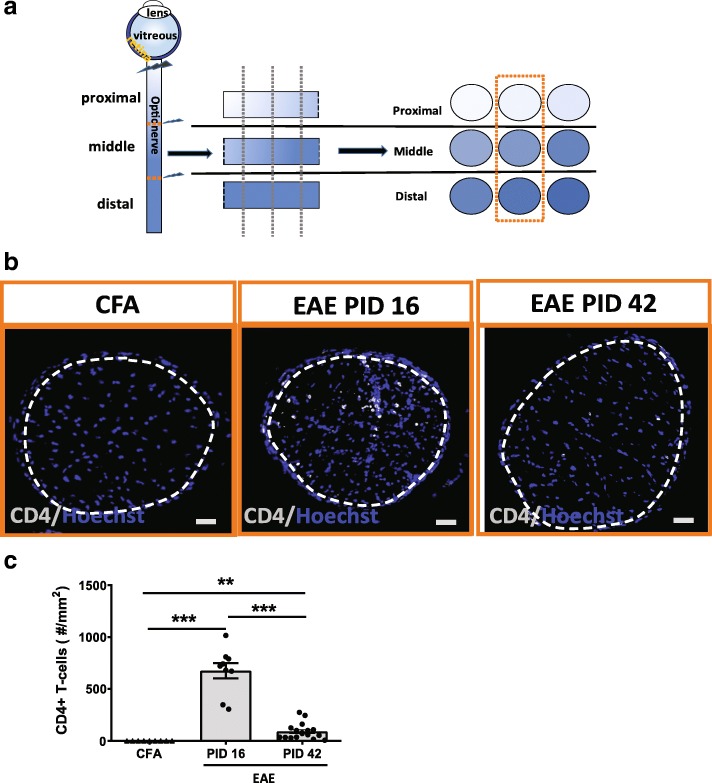
CD4+ T-cell infiltration was prominent in early EAE and diminished in late EAE*.* **a** Schematics for optic nerve sample preparation. Each optic nerve was divided into 3 parts equally, as proximal, middle and distal to the optic nerve head. Each part had 3 regions of interest selected and total of 9 sections for each mouse were stained with interested antibodies. **b** CD4 staining in cross sectioned optic nerve of EAE mouse at PID 16 and PID 42, respectively. CFA group was from control of PID 42 since 2 CFA groups had no difference. White dashed lines delineate the ROI. **c** Quantification of CD4+ T-cells in EAE and CFA control mice. CD4+ T-cells for each animal was based on average of 9 sections from each mouse. Quantification is represented as mean ± SEM. Significance was compared by two-tailed, unpaired Student’s t-test with *P* < 0.05 considered as significant. *** *P* ≤ 0.001. ** *P* ≤ 0.01. Scale bar =50 μm

Since EAE is a T-cell mediated model characterized by myelin reactive T-cell infiltration into the CNS, we examined T-cells in the optic nerve at PID 16 and PID 42. Each optic nerve was divided into 3 parts: proximal, middle and distal to optic nerve head (Fig. 4a and Additional file 4: Figure S4) and a ROI was selected in each optic nerve (Fig. 4b). There were no infiltrating T-cells in the CFA control mice. There was a significantly increased number of infiltrating CD4+ T-cells at PID16 in the optic nerves of EAE mice (*n* = 9) vs CFA control (*n* = 9). The number of CD4+ T-cells was markedly reduced at PID 42 (*n* = 17), but some remained present (Fig. 4b and c).

### Demyelination and axonal injury were evident at early EAE while axonal loss was only seen at late EAE

**Fig. 5:**
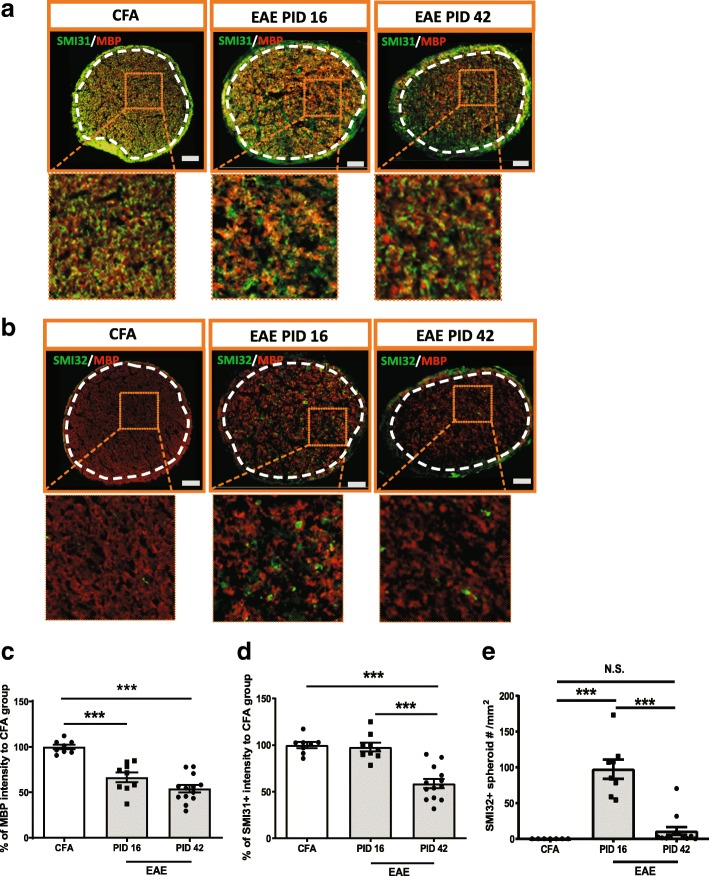
Demyelination and axonal injury were evident at early stage of EAE while axonal loss only was seen at the late stage of EAE. **a** SMI31 and MBP staining in the optic nerve of EAE mice. **b** SMI32 and MBP staining in the optic nerve of EAE mice. **c**-**d** Quantification of MBP and SMI31 as in figure a. **e** Quantification of SMI32 spheroid staining as in figure b. Boxed regions from each images were magnified as shown in figures. White dash line delineated ROI. Quantification is shown as mean ± SEM. Significance was determined by two-tailed, unpaired Student’s t-test with *P* < 0.05 was considered as significant. *** *P* ≤ 0.001. N.S. = no significant difference. Scale bar =50 μm

There was significant myelin loss as revealed by myelin basic protein (MBP) immunohistochemistry in the optic nerves of EAE mice (*n* = 9) vs CFA control animals (*n* = 8) at peak stage of EAE, which persisted into the late stage of EAE (*n* = 13) (Fig. 5a, b and c and Additional file 5: Figure S5a). Although there was no axonal loss, as measured by SMI31^+^ staining, at the early stage of EAE, there was evidence of impaired fast transport as suggested by a significant increase of SMI32^+^ spheroid accumulations in the optic nerve axons of EAE mice at peak stage. At late stage EAE, there was decreased SMI31^+^ and SMI32^+^ staining, consistent with axonal demise (Fig. 5a, b, d, e and Additional file 5: Figure S5b).

### Astrocyte and microglia cells were activated in the optic nerve of EAE

**Fig. 6:**
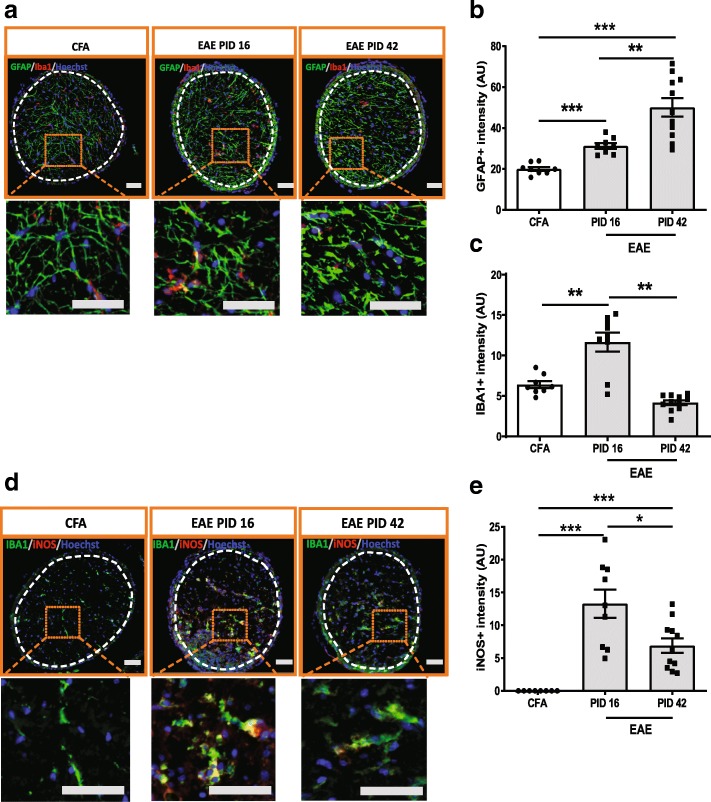
Astrocytes and microglia cells were activated in the optic nerve of EAE. **a** GFAP and IBA1 staining in the optic nerve of EAE mouse. **b** Quantification of GFAP staining in optic nerve. **c** Quantification of IBA1 staining in optic nerve. **d** IBA1 and iNOS double staining in the optic nerve of EAE. **e** Quantification of iNOS staining in optic nerve. Boxed regions from each image were magnified as shown in figures. White dashed lines delineate the ROI. Quantification is shown as mean ± SEM. Significance was determined by two-tailed, unpaired Student’s t-test with *P* < 0.05 consider as significant. * *P* ≤ 0.05, ** *P* ≤ 0.01, *** *P* ≤ 0.001. Scale bar =50 μm

At PID16, astrocytes in the optic nerve of CFA control mice (*n* = 8) were slim with thin processes, whereas in EAE mice (*n* = 9) the astrocytes expressed higher levels of GFAP and these GFAP+ cells exhibited thicker processes than those of CFA controls (Fig. 6a, b and Additional file 6: Figure S6a). At PID42, astrocytes had even higher GFAP levels and thicker processes than those at peak stage, consistent with perpetuation of astrogliosis in late EAE (*n* = 11) (Fig. 6a, b and Additional file 6: Figure S6a). In addition to astrocyte reactivity, microglia were activated as well in peak EAE, as indicated by IBA1 staining. In the CFA control animals (*n* = 8), the microglia in the optic nerve appeared as small cells with ramified morphology. Whereas, in peak EAE mice (*n* = 9), IBA1+ cells appeared activated as compared to those of the CFA controls (Fig. 6a, c and Additional file 6: Figure S6a). These activated microglia also expressed high levels of iNOS in peak EAE (*n* = 9) (Fig. 6d, e and Additional file 6: Figure S6b). At late stage, EAE mice had reduced IBA1 expression, similar to CFA control mice. iNOS expression was reduced at late EAE (*n* = 11); however, the levels were still higher than CFA controls (Fig. 6d, e and Additional file 6: Figure S6b).

**Fig. 7:**
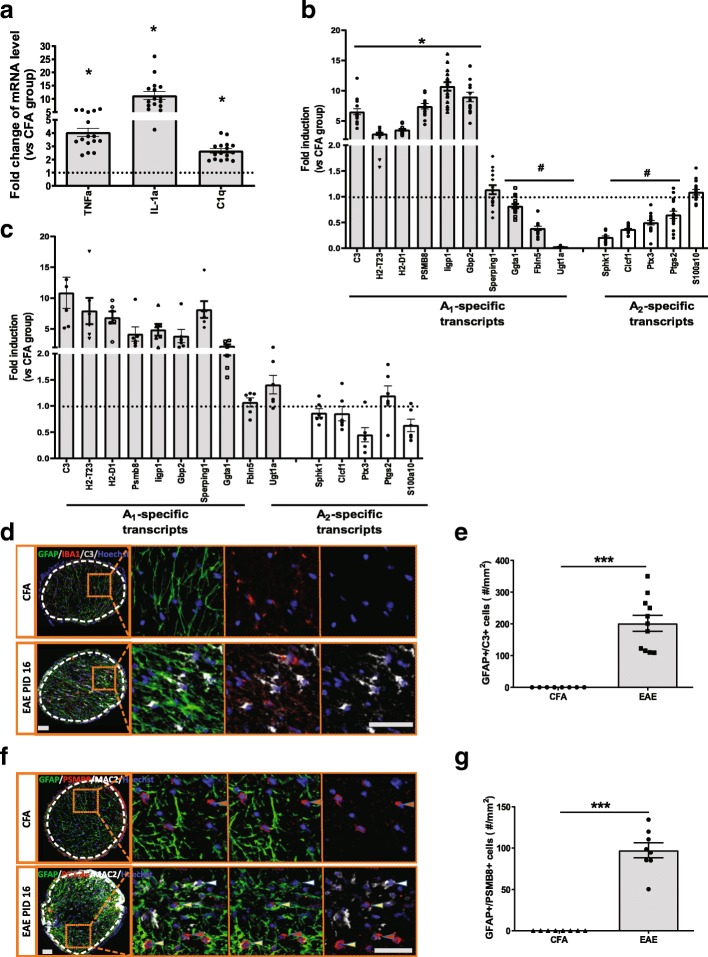
Pro-inflammatory cytokines associated with activated microglia and neurotoxic astrocytes are upregulated in EAE optic nerve. **a** Gene expression of pro-inflammatory cytokines, *TNF-α*, *IL-1α* as well as *C1q* in the optic nerve of EAE mice (n16) at peak stage using qPCR. **b** Astrocyte A1-specific and A2-specific transcripts expression in the optic nerve of EAE mice as previously published. **c** Astrocyte A1-specific and A2-specific transcripts expression in the purified astrocyte from hindbrain of EAE mice (*n* = 16). **d** GFAP, IBA1 and C3 costaining in the optic nerve of EAE mouse. Immunofluorescence staining further confirmed that increased C3 was predominantly expressed in astrocytes and not microglia. **e** Quantification of cells double positive for C3 and GFAP staining (*n* = 8 for CFA; *n* = 11 for EAE). **f** GFAP and PSMB8 co-staining in the optic nerve of EAE mouse. Yellow arrowhead indicated GFAP+/PSMB8+ cell; red arrowhead indicated GFAP+/PSMB8- cell; white arrowhead indicated MAC2+/PSMB8- cell. **g** Quantification of cells double positive for GFAP and PSMB8 staining (*n* = 8 for CFA; *n* = 11 for EAE). Boxed regions from each image were magnified as shown in figures. White dashed lines delineate the ROI. Quantification was represented as mean ± SEM. Significance was determined by two-tailed, unpaired Student’s t-test with *P* < 0.05 consider as significant. * *P* ≤ 0.05, *** *P* ≤ 0.001. Scale bar =50 μm

To follow-up on this finding that astrocytes were persistently activated in the EAE mice, we interrogated their gene expression profile by quantitative PCR (qPCR). A recent publication revealed that a subtype of astrocytes expressing a specific set of genes (A1-transcripts) were neurotoxic whereas A2-transcript expression in astrocytes was associated with release of neurotrophic factors and promotion of neuronal survival. A cocktail of pro-inflammatory cytokines, TNF-α, Il-1α and C1q was sufficient to induce astrocytes to A1 neurotoxic subtype [27]. We extracted total mRNA from the optic nerve of EAE mice at PID 16, during peak inflammation, and examined mRNA levels of these three pro-inflammatory cytokines, tumor necrosis factor- alpha (*TNF-a)*, complement component 1q (*C1q)*, and interleukin-1 alpha (*IL-1α)*. These genes were markedly increased (Fig. 7a). We also confirmed that a number of A1 astrocytes associated genes, including *C3* and *PSMB8*, were upregulated*,* whereas expression of a number of A2 genes, including Cardiotrophin-like cytokine factor 1 (*CLCF1*)*)*, were reduced in optic nerve samples (Fig. 7b). To further confirm that these A1 and A2 transcripts were astrocyte specific, we isolated astrocytes from the hindbrains of EAE mice at PID 16 and measured the panel of transcripts associated with the A1/A2 astrocyte profile, as was recently reported [27]. The results revealed an increase of A1 transcripts and reduction of A2 transcripts in EAE brain as compared to CFA controls (Fig. 7c). Immunofluorescence staining also confirmed increased GFAP+/C3+ double positive glia in the optic nerves of peak stage EAE mice (Fig. 7d and e), which indicated that the astrocytes were skewed to A1 phenotype in EAE. There were also increased numbers of GFAP+/PSMB8+ cells in EAE, further suggesting the presence of neurotoxic A1 astrocytes in EAE (Fig. 7f and g) [27].

## Discussion

Herein, we show that A1 neurotoxic phenotype astrocytes are prevalent in optic nerve tissue and retina, and are associated with subsequent RGC loss, in the most commonly used form of the EAE model induced by MOG _35–55_ peptide in C57/B6 mice. These data corroborate and extend a recent report using ribotag technology to examine gene expression profiles from EAE optic nerve derived astrocytes, in which several A1 genes were highly expressed [17]. The data from this prior report highlight the expression of A1 transcripts including C3, in the optic nerve, and not in the spinal cord. In our study, there was early infiltration of T-cells and associated iNOS producing microglia and astrocytosis in the optic nerve at peak disease (PID 16) of EAE. However, importantly the RGCs were preserved at this time point with no evidence of retinal pathology. At a late stage of EAE, PID 42, the T-cells and microglial responses had clearly waned but astrogliosis persisted and marked RGC loss was observed. The pattern of RGC loss was evenly distributed within the central, middle, and peripheral regions. There was associated decrease in the post-synaptic protein marker, PSD95, and β-III tubulin, which is consistent with RGC and post-synaptic pathology. Although we didn’t find differences in the staining intensity and area of the presynaptic marker, SYP, between EAE and CFA mice, the staining pattern was different in EAE where SYP+ staining was patchy and clustered just below the RGC layer and the IPL; while in the CFA control mice, it was evenly distributed within IPL layer. Preserved SYP may indicate functional compensation as described in models of raised intraocular pressure [32] (Park, 2014, Molecular Brain).

Our findings replicate several previous studies showing RGC loss in both C57/B6 and SJL mice [14, 15, 34, 46, 47]. The timing of RGC loss in the MOG_35–55_ EAE model appears to differ from that reported by another group in which early RGC loss was seen, even before behavioral onset, in rat EAE using rMOG _1–125_ [12]. We cannot exclude the possibility that our approach did not have the sensitivity to detect subtle changes in RGC, but a more likely explanation for the difference is that induction of EAE with rMOG_1–125_ induces both a T cell and an antibody response, and the latter may mediate more rapid and severe disease, as well as species differences between rats and mice.

One of the caveats of examining neurotoxicity in EAE and testing putative neuroprotective compounds is that it is challenging to completely dissociate the effects of peripheral immune cells infiltrating into the CNS from glial cells that may be effectors of secondary neuronal injury. These processes overlap temporally and activated glia facilitate secondary recruitment of peripheral immune cells through release of chemokines. Therefore, the two cannot be completely disassociated. However, a better understanding of the time course and effector profiles of CNS cells in EAE may be important in designing experiments for therapeutic interventions, as well as in interpreting the results. For example, drugs that are predominantly cell trafficking inhibitors are more likely to work early on before peak disease onset, and drugs that target inflammatory monocytes and iNOS producing microglia might be most effective before or during the early but self-limited wave of these cells in EAE between days 8–14 [9]. Therefore, if microglia that produce C1q, TNF-α and IL-1α comprise a subset of these iNOS+ cells then inhibition of these microglial pathways may also have a limited therapeutic window. However, drugs that target activated microglia and subsequent polarization of astrocytes to the neurotoxic A1 phenotype have been described in other models of neurodegeneration and may be useful in as neuroprotective agents for RGC toxicity as occurs in EAE and MS [54]. Alternatively, in a prion model system targeting microglia did not mediate retinal protection [48].

Therapies directly targeting neurotoxic astrocytes could remain effective at later stages of EAE since the astrocytosis appears to persist, unlike the T-cells and microglia. The possibility that RGCs sustain irreversible damage at a certain time between PID 16 and PID 42 could potentially limit the window for neuroprotection in EAE. Further direct mechanistic evidence that neurotoxic astrocytes are indeed an important effector of neurodegeneration in EAE remains to be proven. Early depletion of astrocytes was actually shown to aggravate EAE, presumably through impairing the blood brain barrier and elimination of healthy A2 trophic astrocytes. However, targeting neurotoxic A1 astrocytes without affecting neurotrophic A2 astrocyte during peak disease may protect RGC from loss and other neuronal loss as well [11, 50].

**Fig. 8:**
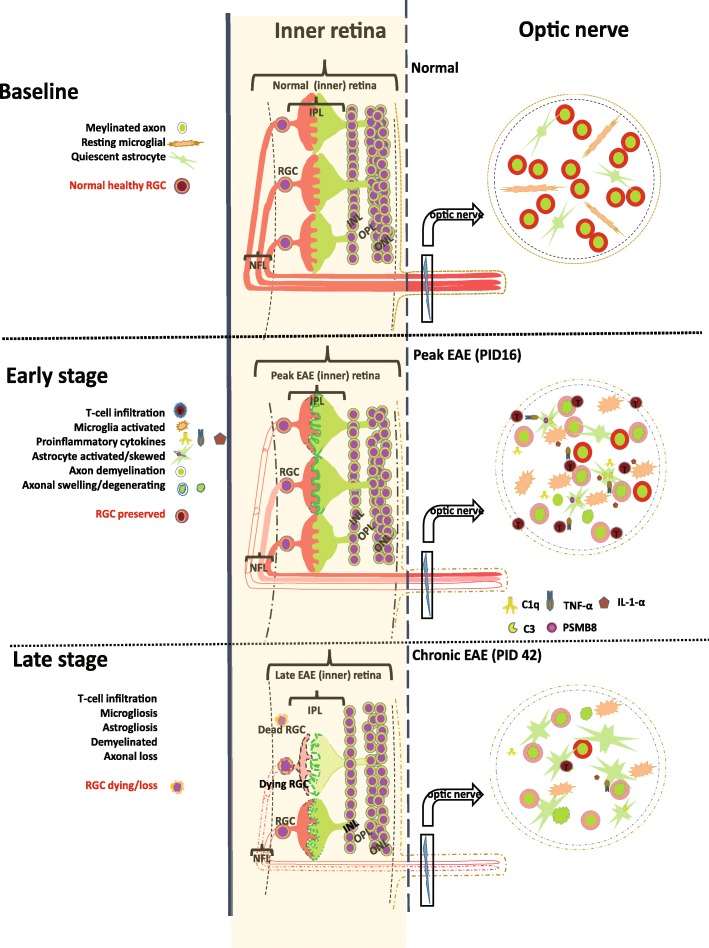
Schematic of glial pathology and neurodegeneration in the retina and optic nerve of EAE mice. In healthy retina, RGCs are layered continuously and there are resting glia (GFAP+ astroglia and microglia). The healthy optic nerve has myelinated axons that are well organized with resting astrocytes and microglia as well. In the early stages of EAE, optic nerve axons were demyelinated in association with infiltrating CD4+ T-cells and activation of microglia. It is thought that activated microglia release cytokines that polarize astrocytes towards a neurotoxic A1 profile associated with neurotoxicity. In the later stages of EAE, post-synaptic proteins and RGCs are depleted. Our findings suggest that EAE recapitulates aspects of MS pathology seen in the anterior visual pathway, and importantly, the time course of events suggests that the MOG _35–55_ EAE model could be used to examine the neuroprotective potential of inhibiting activated microglia or neurotoxic astrocytes at peak disease to prevent late neurodegeneration

Overall, these findings suggest an orchestrated tissue immune response incited by infiltrating immune cells, with a rapid secondary glial response and late neuronal demise (Fig. 8). Importantly, the glial response mirrors the microglial and astroglial findings recently reported with loss of trophic support of glia and adoption of a neurotoxic profile [27]. Thus, we suggest that EAE may be useful to study the glial component of inflammation that occurs in MS and may allow understanding of the CNS cellular mechanisms associated with failed remyelination and neurotoxicity.

There are several limitations to the present work. First, as discussed, these data are mostly associative and do not prove causation of any cell type, especially the neurotoxic astrocytes. For this purpose, we plan to utilize mice with astrocyte specific promoter inducible Cre expression that are crossed with mice in which the target genes of interest have flanking loxp sites to allow for conditional deletion. This approach will allow interrogation of glial effector mechanisms to further elucidate the role of subsets of glia, such as neurotoxic astrocytes, in mediating neuronal injury, and inhibiting myelin repair through suppression of oligodendrocyte precursor cells. Finally, results from EAE may not reflect MS pathophysiology because it models only some aspects of the disease and not the disease itself in which insidious neurodegeneration is a prominent feature [24]. In addition, there are multiple examples of pathways differentially expressed in mice and humans. Further, the EAE model we studied is a monophasic optico-spinal predominant process that in some ways resembles either an acute attack of MS or related optico-spinal diseases moreso than relapsing remitting or progressive MS. Nonetheless, there are distinct advantages to the use of in vivo animal models, and testing of neuroprotective therapies in such a model is a critical step prior to embarking on expensive and potentially risky studies of experimental drugs in humans. EAE has strongly supported the development of several existing drugs for relapsing forms of MS. If we could use the EAE model to examine drugs that target CNS effector mechanisms underlying neuronal injury this would be a major advance given the lack of any true models of progressive disease [24].

## Conclusion

Here we demonstrate a reliable and efficient semi-automatic method of quantification of RGC to monitor neurotoxicity in the anterior visual pathway in the MOG _35–55_ EAE mouse model. EAE mice had significant expression of iNOS+ microglia and A1 neurotoxic astrocytes in the optic nerve followed by RGC loss at the late stage of EAE. Thus, this commonly used EAE mouse model is a useful tool for studying glial mechanisms potentially involved in mediating neurotoxicity, and may be useful for testing neuroprotective agents for MS and other A1 astrocyte related neurodegenerative diseases. The remarkable pathological similarities to specific aspects of MS described here, including the loss of RGCs following acute optic neuritis, and the A1 profile of astrocytes, especially C3 expression, which has been implicated in several recent pathological studies of MS lesions [2, 16, 30, 36, 39, 52], lends support to the idea that the pathogenesis and therapy of glial mediated neurodegeneration may be studied in EAE.

## Additional files

Additional file 1: **Figure S1.** Semi-automated analysis of Brn3a-postive retinal ganglion cell density across whole flat mount retinas. a Whole flat mount retina stained with 3 different antibodies, NeuN, a neuronal specific marker, and RBPMS and Brn3a, RGC specific markers. Scale bar =50 μm. b Comparison of RGC number from automatic and manual count in random selected EAE (*n* = 5, red labels) and wild-type mice (*n* = 6, blue labels). c Comparison of RGC density in subregions of whole retina from automatic and manual count in random selected EAE (*n* = 5) and wild-type mice (*n* = 6). Automated analysis accurately reflected regional differences. d The RGC density between left and right retinas of the same mouse were comparable. e Comparision of RGC number counted by different persons. N.S. = no significant difference. (PDF 890 kb)

Additional file 2: **Figure S2.** RGC numbers in PID 16 EAE and in CFA control mice vs healthy controls. a Brn3a staining at PID16. b Quantification of RGC number at PID 16. c RGC number of central retina at early stage EAE (PID16). d RGC number of middle retina at early stage EAE (PID16). e RGC number of peripheral retina at early stage EAE (PID16). f RGC density of naïve and CFA control mice. g There were no difference in BS between female and male mice. N.S. = no significant difference. Red scale bar =1 mm. White scale bar =20 μm. (PDF 519 kb)

Additional file 3: **Figure S3.** Synaptic density marker staining and IBA1+ cell numbers in inner retina of EAE mice at PID42. a Quantification of PSD-95 (a) and SYP (b) staining by positive staining area in the inner retina of EAE mouse (*n* = 15) and CFA control (*n* = 9). c IBA1+ cell number in inner retina of EAE mice. Quantification is represented as mean ± SEM. Significance was determined by two-tailed, unpaired Student’s t-test with *P* < 0.05 considered signficant. ** *P* ≤ 0.01. N.S. = no significant difference. (PDF 72 kb)

Additional file 4: **Figure S4.** CD4+ T-cell staining in the optic nerve of EAE and CFA control mice. a CD4 staining in different degion of cross sectioned optic nerve of EAE mouse at PID 16 and PID 42, respectively. CFA group was from control of PID 42 since 2 CFA groups had no difference. Scale bar =50 μm. (PDF 388 kb)

Additional file 5: **Figure S5.** SMI31, MBP and SMI32 staining in the optic nerve of EAE and CFA control mice. a SMI31 and MBP staining in different region of optic nerve of in EAE and CFA control mice at PID16 and PID42, respectively. b SMI32 and MBP staining in different region of optic nerve of EAE and CFA control mice at PID16 and PID42, respectively. CFA group was from control of PID 42. Scale bar =50 μm. (PDF 4950 kb)

Additional file 6: **Figure S6.** GFAP and IBA1 staining in the optic nerve of EAE and CFA control mice. a GFAP and IBA1 staining in different region of optic nerve of in EAE and CFA control mice at PID16 and PID42, respectively. b IBA1 and iNOS staining in different region of optic nerve of EAE and CFA control mice at PID16 and PID42, respectively. CFA group was from control of PID 42. Scale bar =50 μm. (PDF 5320 kb)

Additional file 7: RGC count algorithm (MATLAB based). (M 10 kb)

## Data Availability

The datasets used and/or analyzed during the current study are available from the corresponding author on reasonable request. The semi-automated RGC counting algorithm is also available either by request to corresponding author or can be uploaded into the journal website for free download.

## Abbreviations

Brn3a: Brain-specific homeobox protein 3A
BS: Behavioral score
*C1q*: Complement component 1q
*C3*: Complement component 3
CFA: Complete Freund’s adjuvant
*CLCX1*: Chemokine (C-X-C motif) ligand 1
CNS: Central nervous system
EAE: Eperimental autoimmune encephalomyelitis
*GBP2*: Interferon-induced guanylate-binding protein 2
GCL: Ganglion cell layer
GFAP: Glial Fibrillary Acidic Protein
IBA1: Ionized calcium-binding adapter molecule 1
*IL-1α*: Interleukin-1 alpha
iNOS: Inducible nitric oxide synthase
IPL: Inner plexiform layer
MOG: Myelin-oligodendrocyte glycoprotein
MS: Multiple sclerosis
OCT: Optical coherence tomography
OCT: Optimal Cutting Temperature compound
OPL: Outer plexiform
PBS: Phosphate Buffered Saline
PFA: paraformaldehyde
PID: Post-immunization day
PSD95: Postsynaptic density-95
*PSMB8*: Proteasome subunit beta type-8
RBPMS: RNA-binding protein with multiple splicing
RGC: Retinal ganglion cell
SYP: Synaptophysin
*TNF-a*: Tumor necrosis factor- alpha
Tuj: β-III-tubulin
