# Supplementary figures and images for: Glial pathology and retinal neurotoxicity in the anterior visual pathway in experimental autoimmune encephalomyelitis
Source: Acta Neuropathol Commun. 2019 Jul 31;7:125. doi: 10.1186/s40478-019-0767-6 (PMC6670238; doi:10.1186/s40478-019-0767-6)

Figure S1.

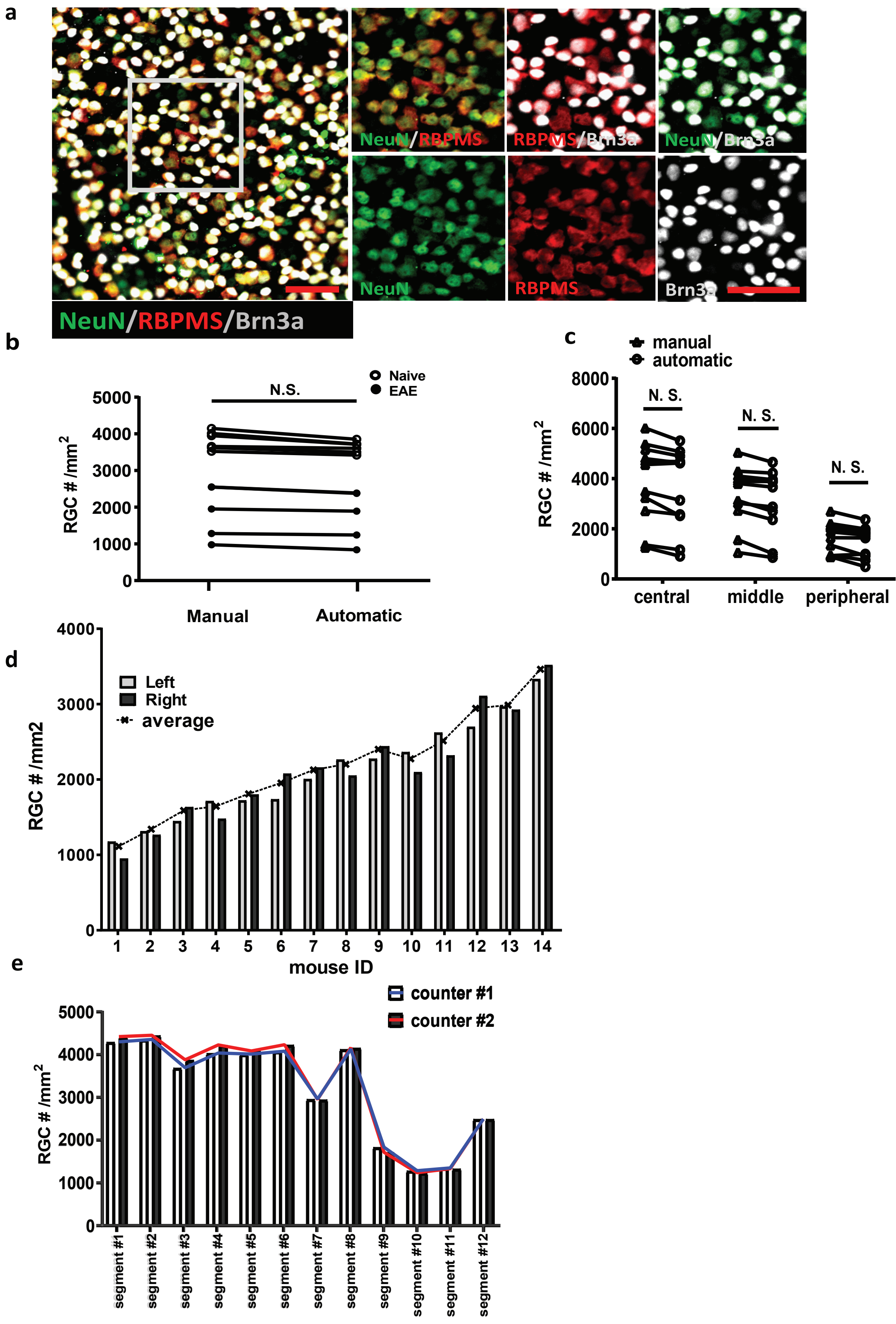

Supplement: Supplementary file 1 — Figure S1. Semi-automated analysis of Brn3a-postive retinal ganglion cell density across whole flat mount retinas. a Whole flat mount retina stained with 3 different antibodies, NeuN, a neuronal specific marker, and RBPMS and Brn3a, RGC specific markers. Scale bar =50 μm. b Comparison of RGC number from automatic and manual count in random selected EAE (n = 5, red labels) and wild-type mice (n = 6, blue labels). c Comparison of RGC density in subregions of whole retina from automatic and manual count in random selected EAE (n = 5) and wild-type mice (n = 6). Automated analysis accurately reflected regional differences. d The RGC density between left and right retinas of the same mouse were comparable. e Comparision of RGC number counted by different persons. N.S. = no significant difference. (PDF 890 kb) [file 40478_2019_767_MOESM1_ESM.pdf]

Figure S2.

a

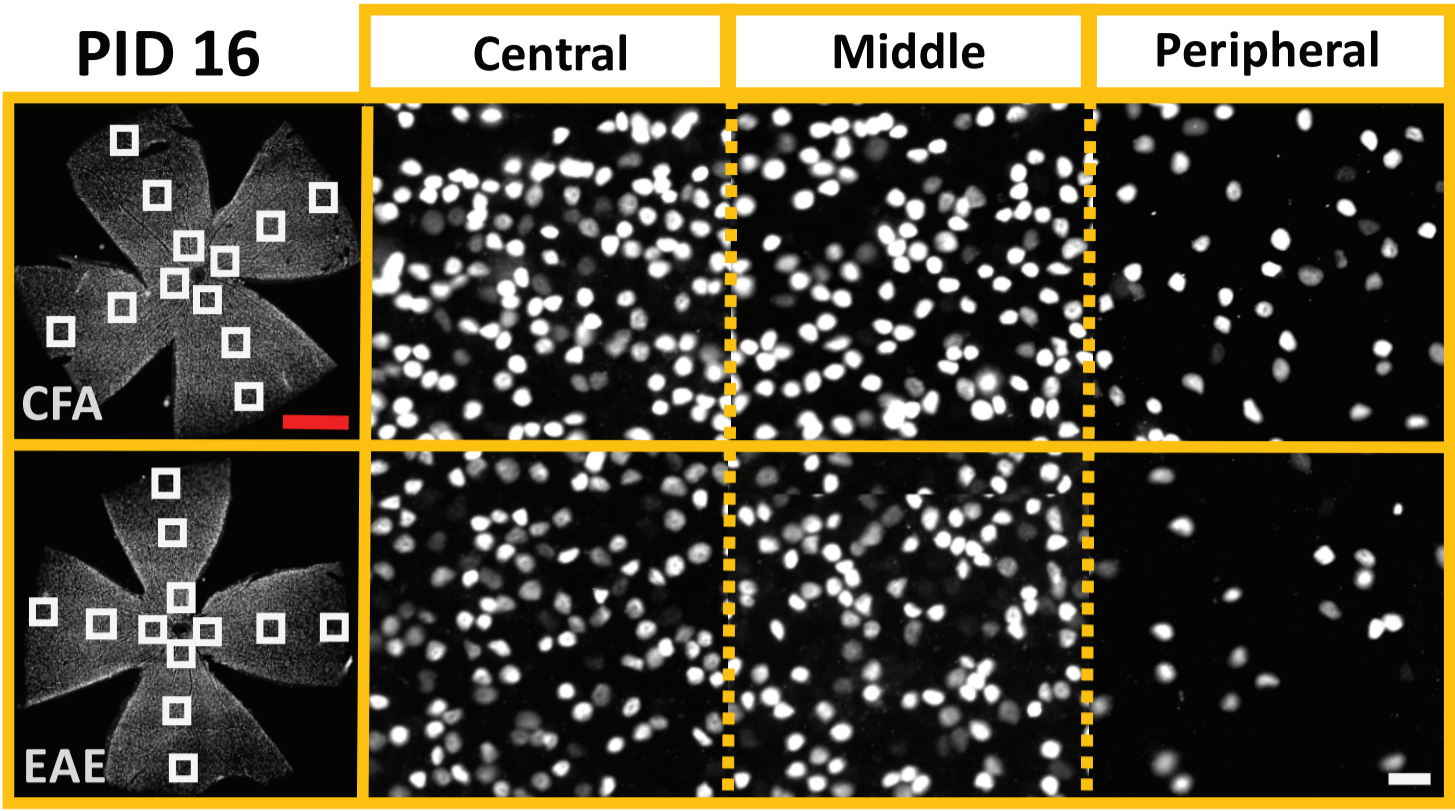

b

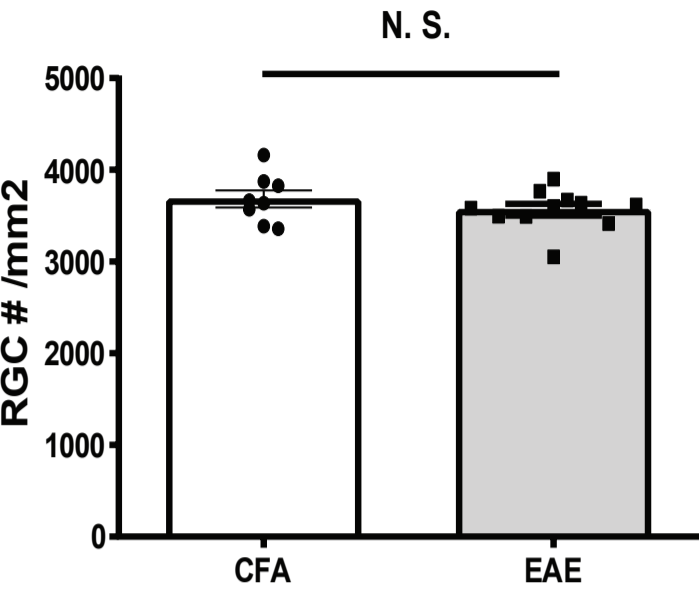

c

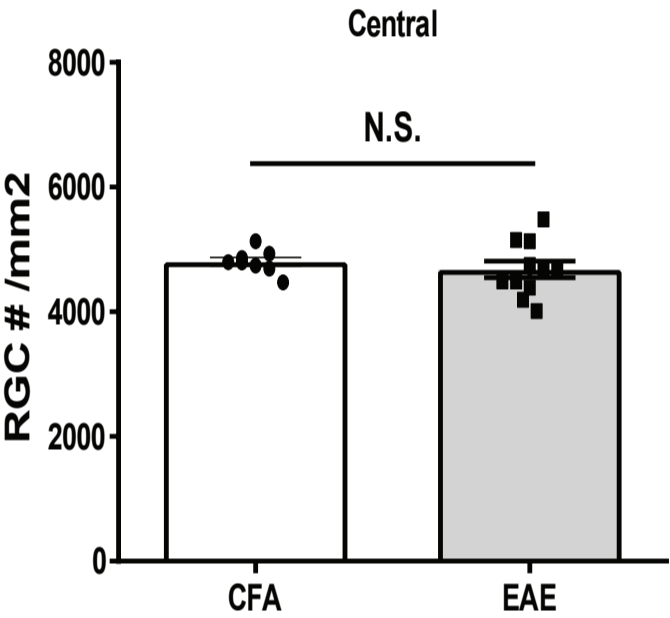

d

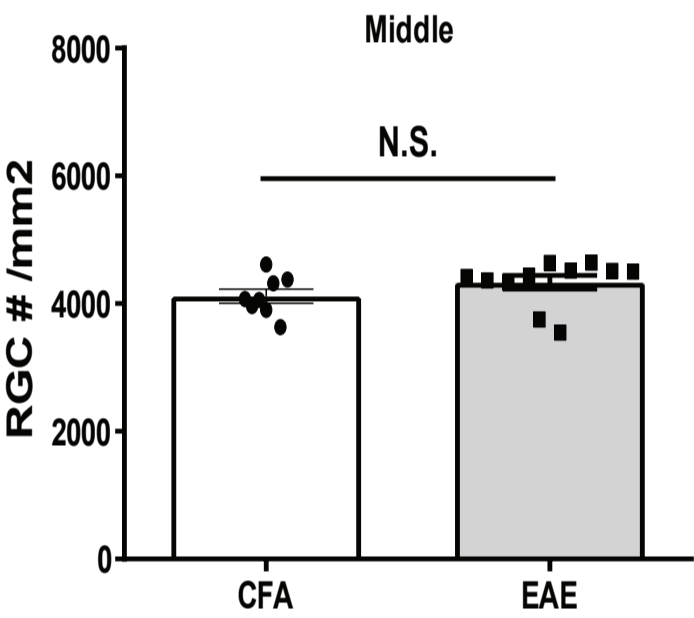

e

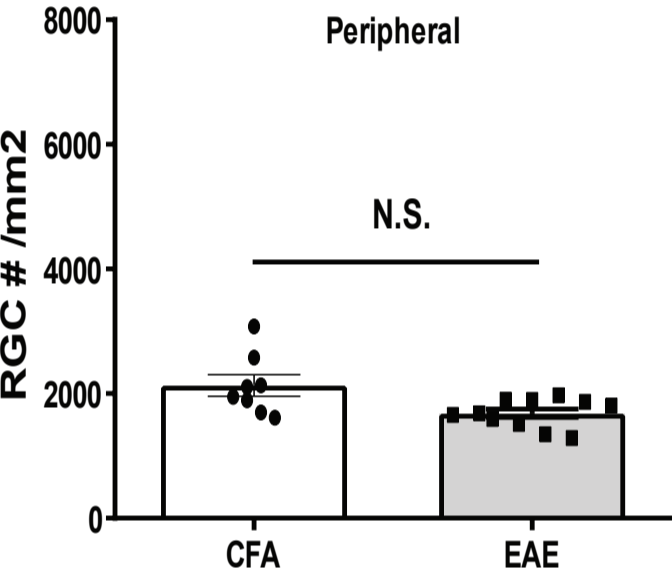

f

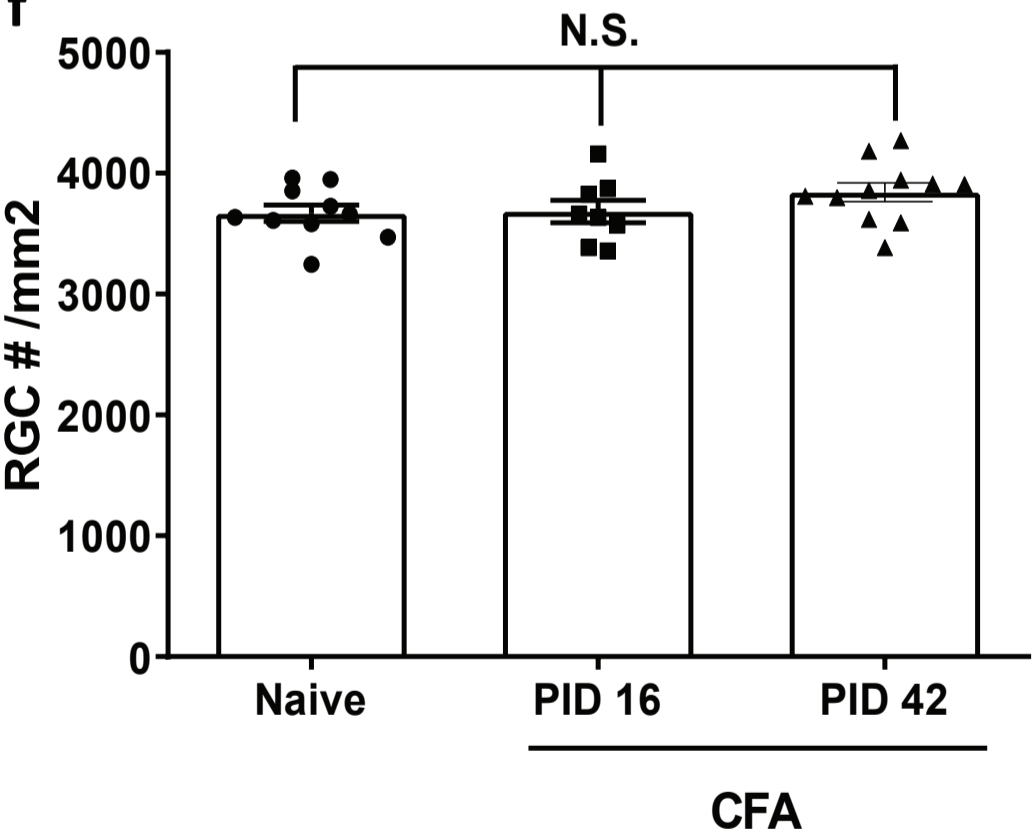

g

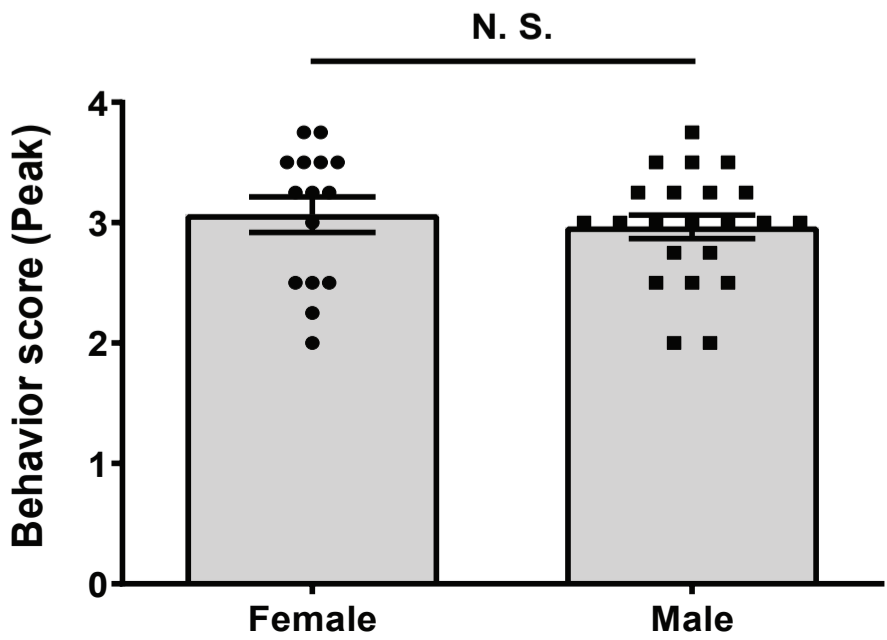

Supplement: Supplementary file 2 — Figure S2. RGC numbers in PID 16 EAE and in CFA control mice vs healthy controls. a Brn3a staining at PID16. b Quantification of RGC number at PID 16. c RGC number of central retina at early stage EAE (PID16). d RGC number of middle retina at early stage EAE (PID16). e RGC number of peripheral retina at early stage EAE (PID16). f RGC density of naïve and CFA control mice. g There were no difference in BS between female and male mice. N.S. = no significant difference. Red scale bar =1 mm. White scale bar =20 μm. (PDF 519 kb) [file 40478_2019_767_MOESM2_ESM.pdf]

**Figure S3.**

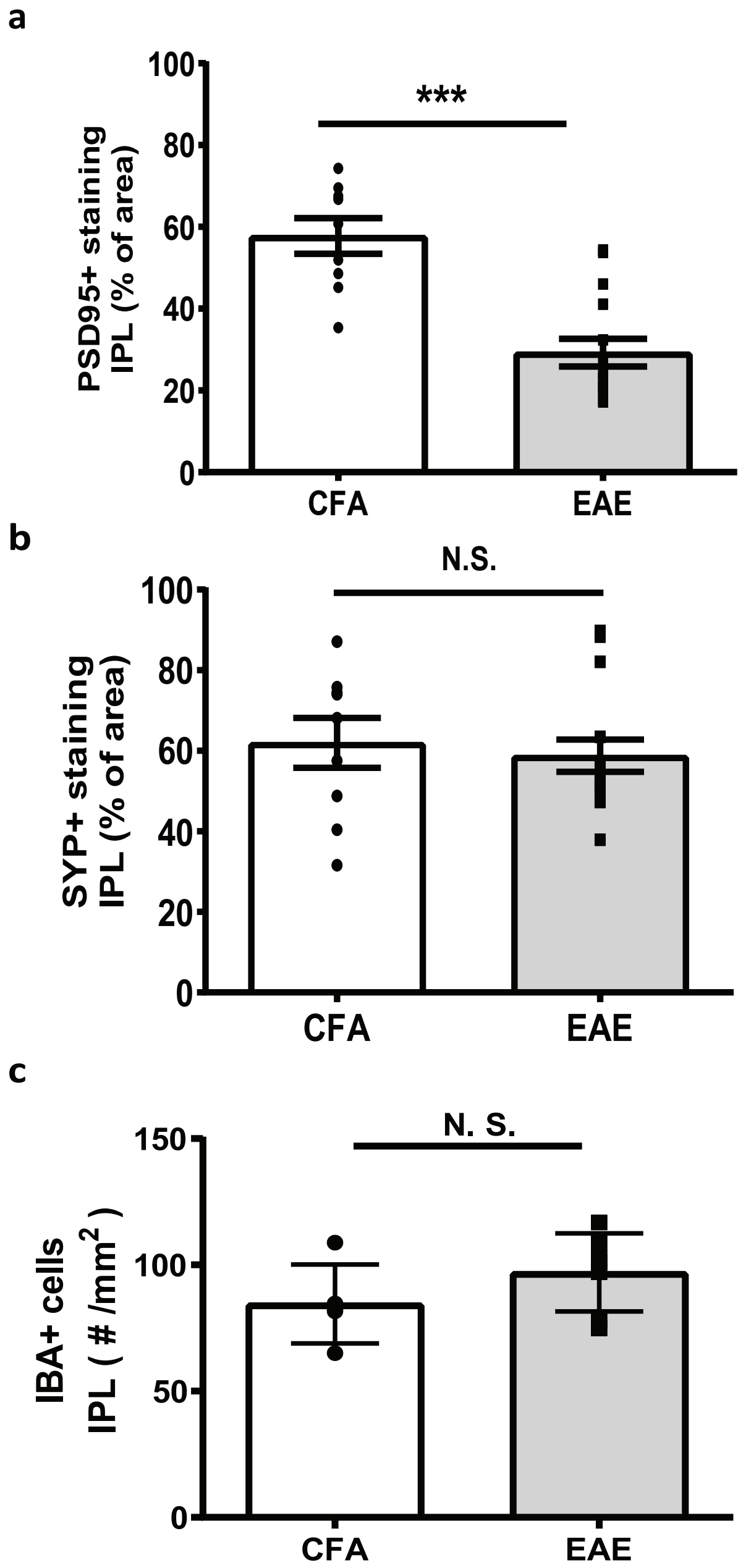

Supplement: Supplementary file 3 — Figure S3. Synaptic density marker staining and IBA1+ cell numbers in inner retina of EAE mice at PID42. a Quantification of PSD-95 (a) and SYP (b) staining by positive staining area in the inner retina of EAE mouse (n = 15) and CFA control (n = 9). c IBA1+ cell number in inner retina of EAE mice. Quantification is represented as mean ± SEM. Significance was determined by two-tailed, unpaired Student’s t-test with P < 0.05 considered signficant. ** P ≤ 0.01. N.S. = no significant difference. (PDF 72 kb) [file 40478_2019_767_MOESM3_ESM.pdf]

Figure S4.  
a

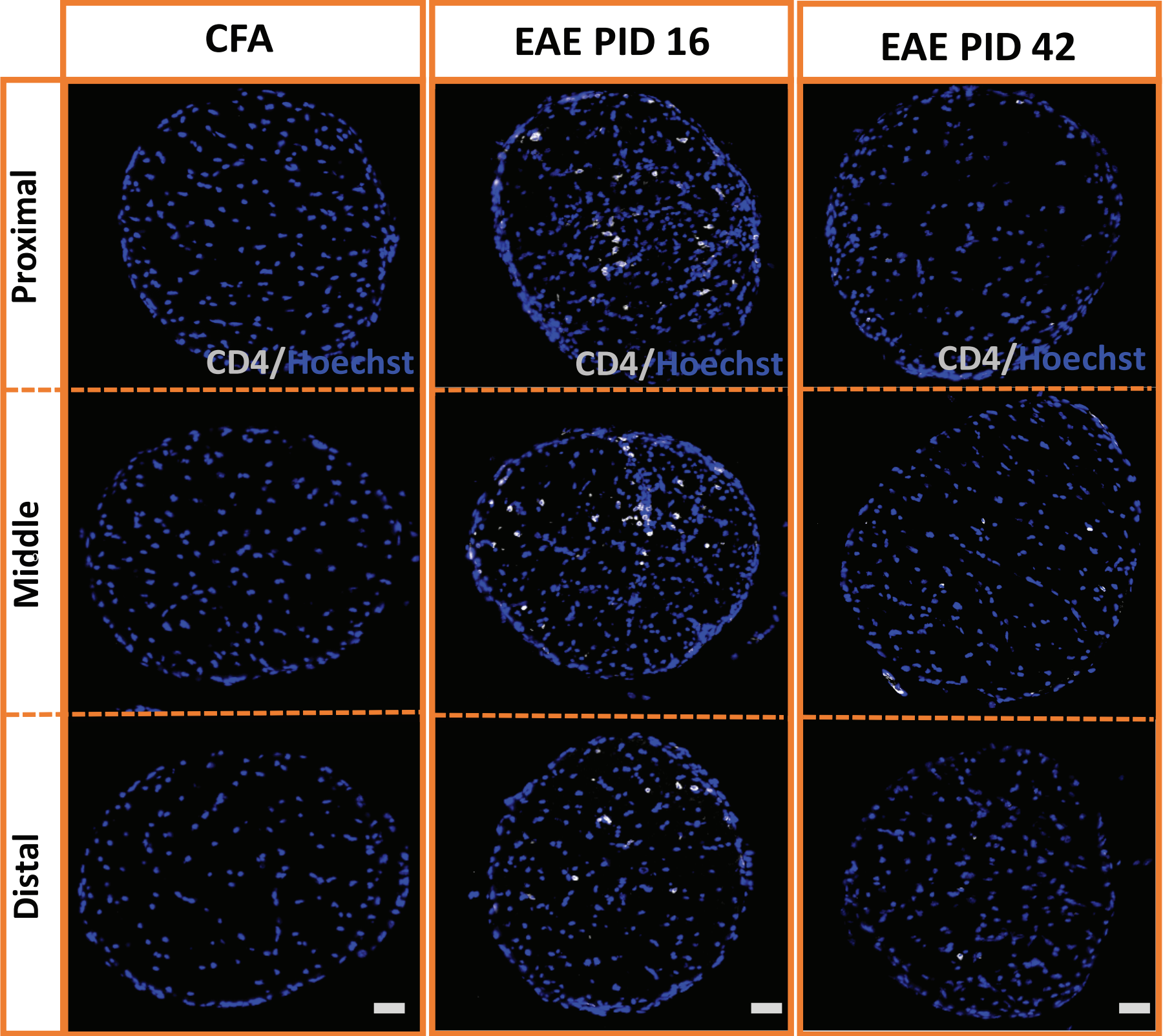

Supplement: Supplementary file 4 — Figure S4. CD4+ T-cell staining in the optic nerve of EAE and CFA control mice. a CD4 staining in different degion of cross sectioned optic nerve of EAE mouse at PID 16 and PID 42, respectively. CFA group was from control of PID 42 since 2 CFA groups had no difference. Scale bar =50 μm. (PDF 388 kb) [file 40478_2019_767_MOESM4_ESM.pdf]

Figure S5.

a

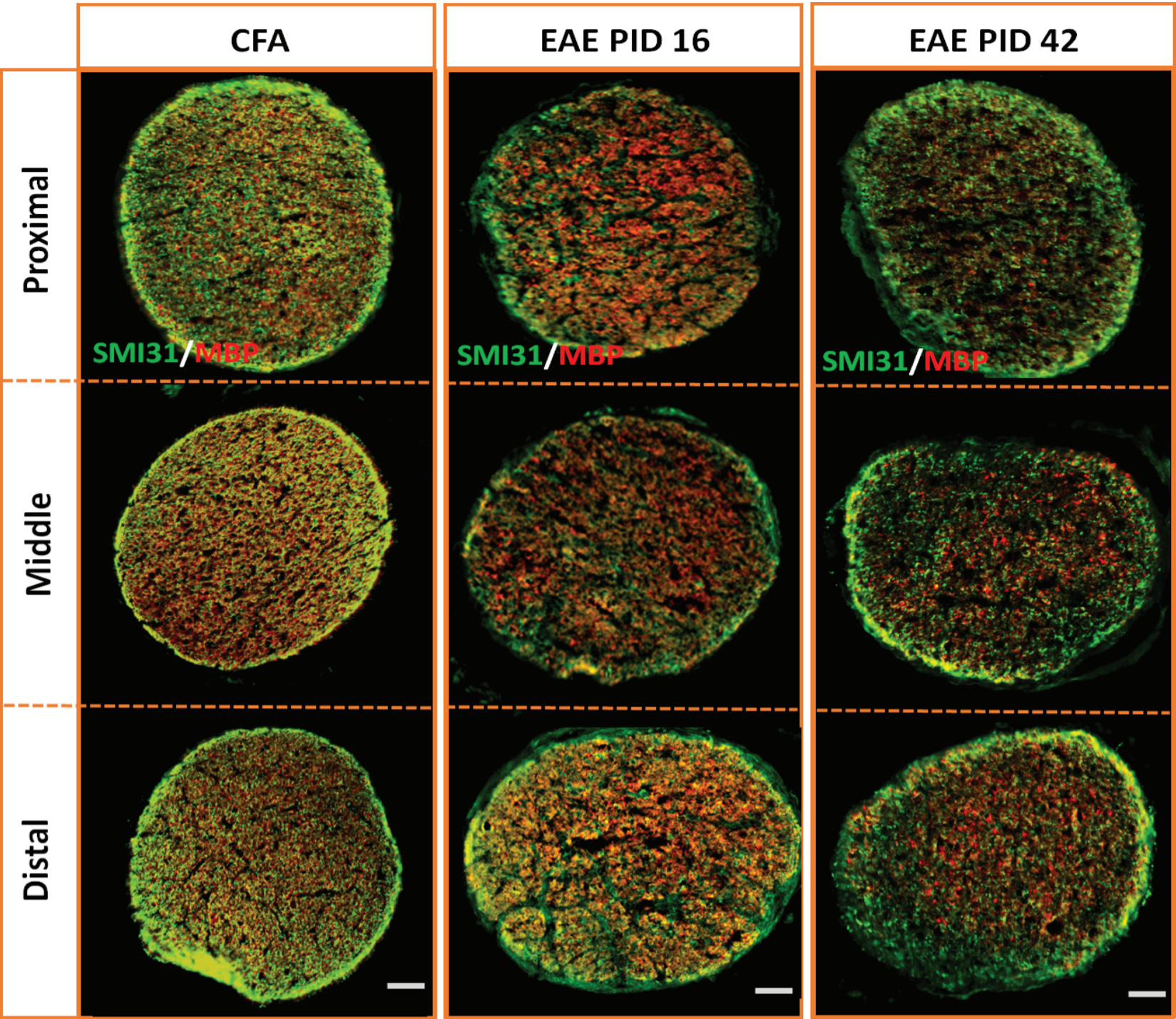

b

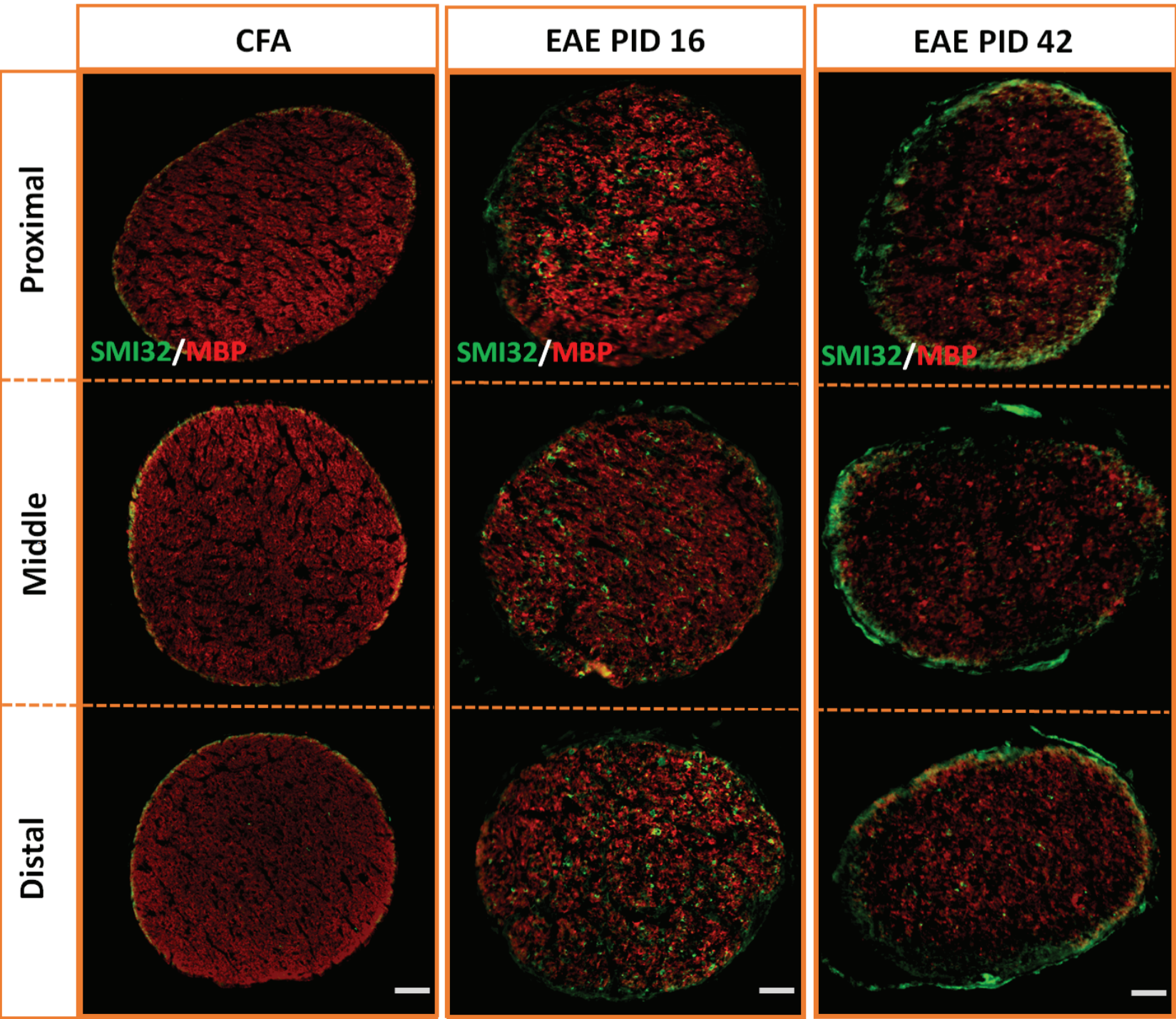

Supplement: Supplementary file 5 — Figure S5. SMI31, MBP and SMI32 staining in the optic nerve of EAE and CFA control mice. a SMI31 and MBP staining in different region of optic nerve of in EAE and CFA control mice at PID16 and PID42, respectively. b SMI32 and MBP staining in different region of optic nerve of EAE and CFA control mice at PID16 and PID42, respectively. CFA group was from control of PID 42. Scale bar =50 μm. (PDF 4950 kb) [file 40478_2019_767_MOESM5_ESM.pdf]

Figure S6.

a

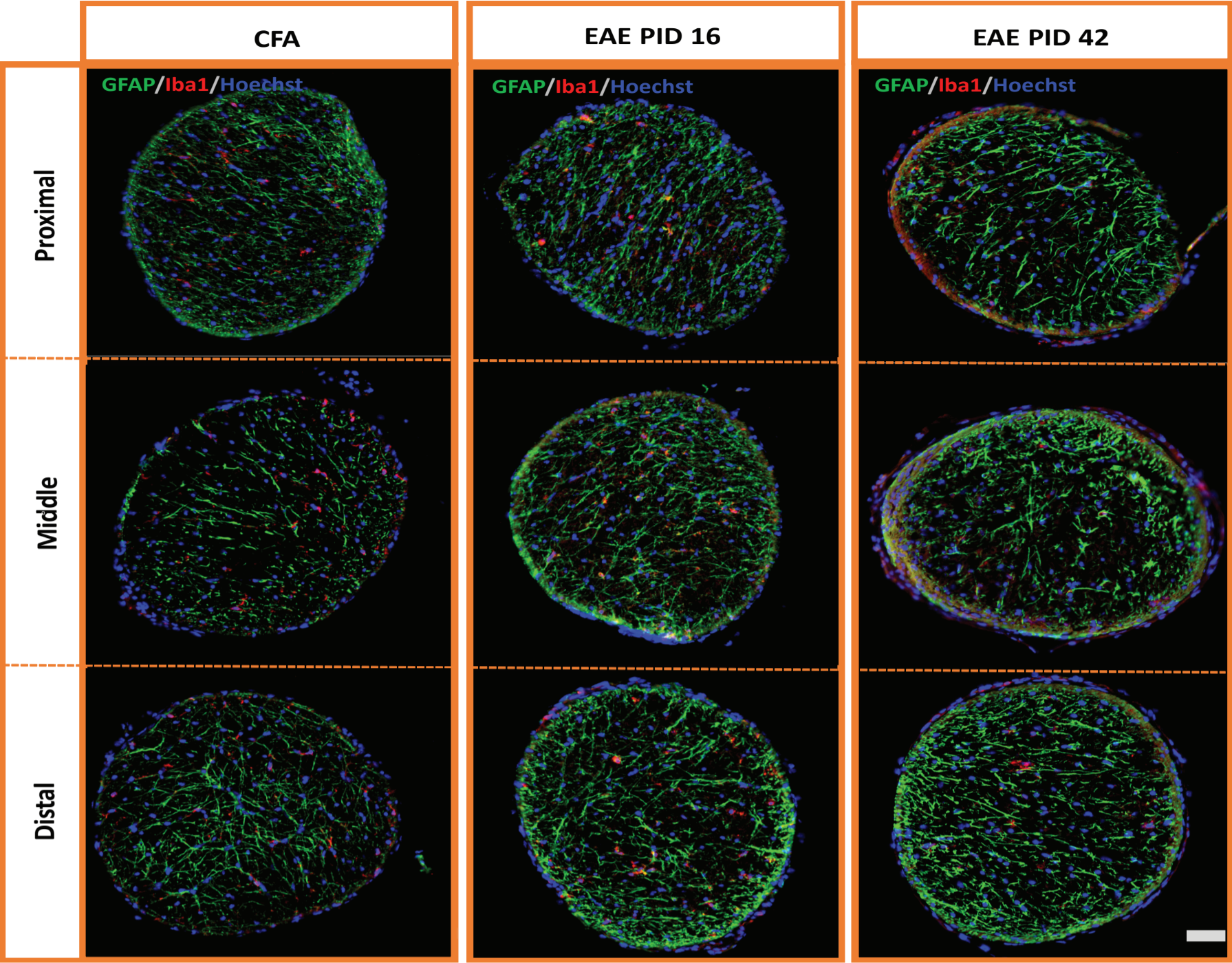

b

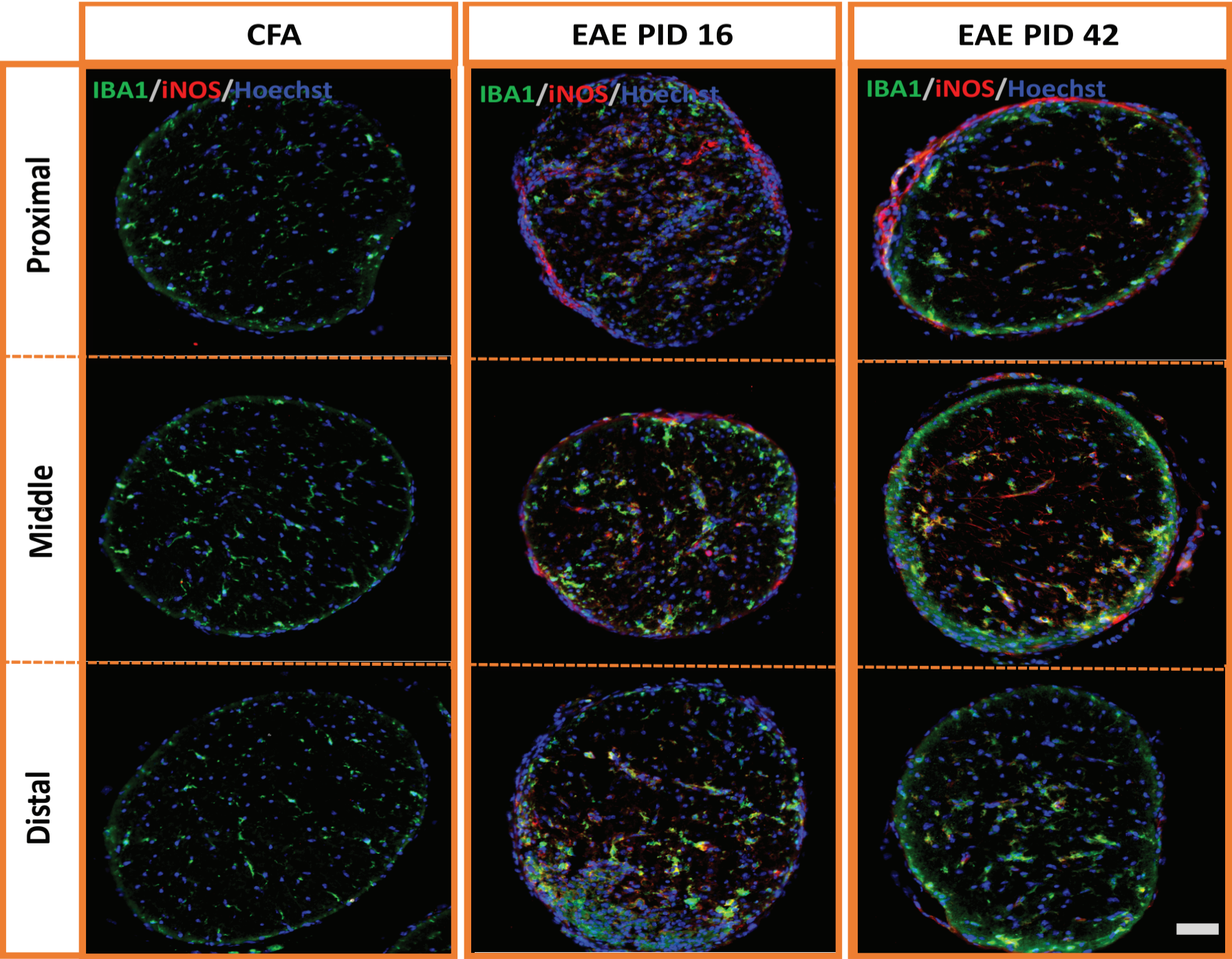

Supplement: Supplementary file 6 — Figure S6. GFAP and IBA1 staining in the optic nerve of EAE and CFA control mice. a GFAP and IBA1 staining in different region of optic nerve of in EAE and CFA control mice at PID16 and PID42, respectively. b IBA1 and iNOS staining in different region of optic nerve of EAE and CFA control mice at PID16 and PID42, respectively. CFA group was from control of PID 42. Scale bar =50 μm. (PDF 5320 kb) [file 40478_2019_767_MOESM6_ESM.pdf]
